# Engineered GM1 Intersects Between Mitochondrial and Synaptic Pathways to Ameliorate ALS Pathology

**DOI:** 10.1002/advs.202514128

**Published:** 2026-01-05

**Authors:** Federica Pilotto, Tristan Dellazizzo Toth, Silvano Bond, Alexander Schmitz, Rim Diab, Sara Y. Ngo Tenlep, Brian Mooney, Silvia Erni, Martina Schobesberger, Olivier Scheidegger, Camille Peitsch, Smita Saxena

**Affiliations:** ^1^ Department of Physical Medicine & Rehabilitation University of Missouri Columbia Missouri USA; ^2^ NextGen Precision Health University of Missouri Columbia Missouri USA; ^3^ Institut Neuromyogène, Pathophysiology and genetics of the neuron and muscle Inserm U1315 CNRS Université Claude Bernard Lyon I Lyon France; ^4^ Division of Biochemistry University of Missouri Columbia Missouri USA; ^5^ Charles W Gehrke Proteomics Center University of Missouri Columbia Missouri USA; ^6^ InnoMedica Schweiz AG Bern Switzerland; ^7^ Department of Neurology Inselspital University Hospital Bern Switzerland

**Keywords:** amyotrophic lateral sclerosis (ALS), C9ORF72, ER‐mitochondria contacts, GM1, mitochondrial dysfunction, nanoliposomal formulation, proteomics, SOD1‐G93A, synaptic dysfunction

## Abstract

Amyotrophic Lateral Sclerosis (ALS) is a progressive and fatal condition marked by the degeneration of motor neurons. ALS has been linked to numerous genes with diverse biological roles, reflecting a highly intricate and multifaceted disease process. This diversity poses significant challenges in developing universally effective and bioavailable treatments. Advancing therapeutic strategies require uncovering molecular pathways that are major drivers of ALS. We conducted proteomic analyses of human iPSC‐derived motor neurons carrying *C9ORF72* mutations, alongside spinal ventral horns from mice with pathogenic *C9orf72*‐mutations. This cross‐species approach revealed disruptions in synaptic vesicle release, endoplasmic reticulum (ER) and mitochondrial stress responses as conserved ALS pathogenic mechanisms. Disease progression was associated with accumulation of cytotoxic protein aggregates and oxidative stress. We analyzed the potential of GM1, an established neuroprotective molecule, to reverse these pathogenic features. To enhance the pharmacokinetics of GM1, we developed Talineuren (TLN), a nanoliposome‐based formulation of the active pharmaceutical ingredient GM1 ganglioside that improves its bioavailability. GM1 stabilized mitochondrial Ca^2^⁺ handling, improved energy metabolism, and alleviated ER stress, preventing protein aggregation and restoring cellular proteostasis and counteracted behavioral deficits in *C9orf72* and *SOD1‐G93A* mouse models. Together, these findings underscore the central, convergent role for cellular disruptions in ALS and position TLN as a promising therapeutic candidate.

## Graphical abstract

Amyotrophic Lateral Sclerosis (ALS) is a fatal neurodegenerative disease driven by genetic and molecular disruptions affecting energy balance, protein homeostasis, and stress responses in nerve cells. Studies using human and rodent models identified convergent defects in mitochondria and synaptic function. GM1‐based nanotherapy, “Talineuren” restored cellular health and motor function, showing strong therapeutic promise.

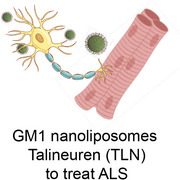

## 1 Introduction

Neurodegenerative disorders such as amyotrophic lateral sclerosis (ALS) are characterized by the progressive degeneration of upper and lower motor neurons, leading to muscle weakness, paralysis, and ultimately respiratory failure. Despite intensive research, ALS remains incurable. While most cases are sporadic, a hexanucleotide G_4_C_2_ repeat expansion in the *C9ORF72* gene is the most common genetic cause of both familial and sporadic forms of ALS [1, 2]. The pathophysiology of *C9ORF72*‐ALS involves a convergence of dysregulated cellular processes, including oxidative stress, mitochondrial dysfunction, chronic endoplasmic reticulum (ER) stress, neuroinflammation, and the accumulation of toxic dipeptide repeat proteins (DPRs) produced via repeat‐associated non‐AUG (RAN) translation. These pathogenic DPRs drive neuronal dysfunction and degeneration through their aggregation and diverse cytotoxic mechanisms [3, 4, 5, 6, 7, 8, 9, 10]. Thus, the multifactorial and interconnected nature of these mechanisms presents a substantial challenge for the development of effective therapies for ALS.

The delivery of molecules to the brain parenchyma remains a major hurdle. Many molecules that show promising results in preclinical studies, significantly improving disease conditions, fail to efficiently cross the blood‐brain barrier (BBB) in humans. This limitation presents a significant obstacle in addressing devastating neurodegenerative diseases. Nanotechnologies are emerging as a promising strategy for tackling neurodegenerative diseases as well as cancer therapies [11, 12, 13]. Nanoparticles such as liposomes, polymeric micelles and nanogels are small in size, have surface functionalization capabilities, and the ability to traverse biological barriers like the BBB [14, 15]. Unlike conventional therapies, nanoparticles can be engineered to provide targeted delivery of therapeutic agents, reducing systemic side effects and increasing drug bioavailability at the site of neuronal damage. Furthermore, nanoparticle platforms can be tailored to release active molecules in response to specific triggers in the microenvironment, such as pH or oxidative stress, enhancing its therapeutic efficacy [14, 16, 17]. This makes them particularly advantageous for treating disorders like ALS, where targeted interventions are critical to slowing disease progression.

GM1 ganglioside, a naturally occurring glycosphingolipid (GSL) enriched in neuronal membranes, has attracted considerable interest in its neuroprotective properties. It plays a critical role in maintaining membrane integrity, modulating signal transduction, and supporting synaptic plasticity [18, 19, 20, 21, 22]. In *SOD1‐G93A* ALS mice, GSL metabolism becomes progressively dysregulated across disease stages, showing a biphasic pattern with an early strong reduction in key GSLs such as GM1, sphingomyelin and cerebrosides, followed by late‐stage accumulation of many GSLs including GM1. The initial reduction in GM1 during disease onset and progression indicates widespread disruption of GSL metabolism as neurodegeneration advances [23, 24, 25]. Notably, this early reduction in GM1 levels indicated a premature reduction in crucial neuroprotective pathways and GM1 accumulation in end stage‐*SOD1‐G93A* mice and in human postmortem tissue suggests a terminal compensatory response to slow disease progression [23]. Importantly, infusion of ganglioside GM3 delayed disease progression in *SOD1‐G93A* mice, highlighting the therapeutic potential of gangliosides for ALS [23]. We thus hypothesized that GM1 supplementation may also counteract ALS‐associated deficits. While recent studies have demonstrated the protective effects of GM1 against glutamate toxicity in cultured *SOD1‐G93A* neurons [26], human clinical trials have yielded inconclusive results likely due to underdosing and the use of mixed ganglioside formulations [27, 28]. Additional barriers such as poor pharmacokinetics, limited BBB permeability, and rapid clearance have hindered its therapeutic development. However, recent Parkinson's disease (PD) clinical trials confirm GM1's safety at higher doses and its potential as both symptomatic and disease‐modifying therapy [29, 30, 31].

To overcome the pharmacokinetic limitations of GM1 and comprehensively assess its neuroprotective mechanism and therapeutic potential in ALS, we developed Talineuren (TLN), a nanoliposome‐based formulation of the active pharmaceutical ingredient GM1 ganglioside designed to enhance GM1 bioavailability and enable targeted, controlled delivery to vulnerable neuronal populations. TLN was evaluated in monogenic mouse models of ALS, specifically *C9ORF72* and *SOD1‐G93A*, which recapitulate key aspects of human disease. The mode of action of TLN was assessed by proteomic profiling of ALS patient iPSC‐derived motor neurons (iMNs) and symptomatic *C9‐500* animals, which revealed that TLN treatment restored homeostasis in pathways linked to mitochondrial function, ER stress, and synaptic integrity. At the organelle level, GM1 stabilized mitochondrial function by ameliorating Ca^2+^ uptake, and supporting energy metabolism. It also mitigated inflammation, ER stress, thereby preventing maladaptive protein aggregation and restoring proteostasis. Through these multifaceted cellular actions, TLN emerges as a central regulator of neuronal integrity and resilience under pathological ALS conditions. These molecular benefits translated into significant neuroprotection in vivo, as demonstrated by improved histopathology and behavioral outcomes in response to TLN in both *C9‐500* and *SOD1‐G93A* models. Cross‐species comparative proteomics further identified conserved molecular and cellular targets of TLN between human and murine systems, strengthening the translational relevance of our findings. Collectively, these data position TLN as a promising therapeutic candidate for ALS and related neurodegenerative disorders, warranting advancement toward clinical evaluation.

## 2 Results

### 2.1 A Nanoparticle‐Based Approach for Efficient Targeting and Uptake of GM1 in the CNS

**FIGURE 1:**
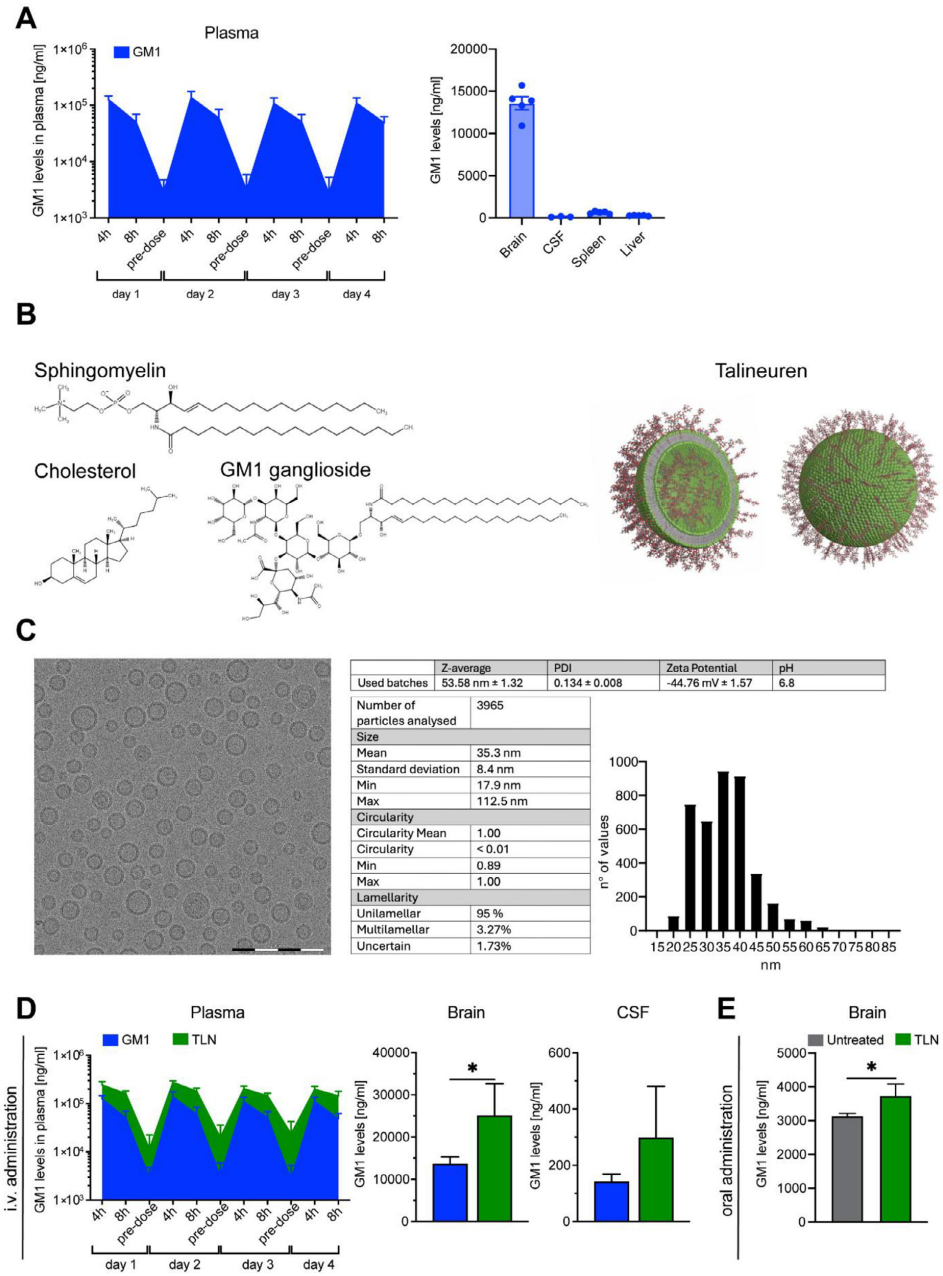
TLN can efficiently deliver GM1 into the brain parenchyma. (A) Left: Free non‐liposomal GM1 was evaluated and compared following repeated i.v. injection in CD‐1 rats. Right: GM1 biodistribution levels evaluated in brain, CSF, spleen and liver after i.v. administration of TLN at a dose of 12.3 mg/kg. Note the higher GM1 expression in the brain reflecting endogenous GM1 expression. N=5 rats/group. (B) TLN is composed of sphingomyelin and cholesterol while GM1 Ganglioside is inserted into the membrane as represented in the 3D reconstruction. (C) Representative cryogenic electron transmission microscopy (cryo‐TEM) image of a representative batch of TLN. Majority of the particles are unilamellar, while a smaller subpopulation of particles displayed multilamellar conformations (3.27 %). N=2241 liposomes analyzed. Scale bar: 500 nm. (D) TLN biodistribution levels evaluated in brain and CSF after i.v. administration. Note a twofold increase in GM1 levels in the brain of rats treated with TLN compared to those receiving free GM1 Unpaired t‐test, t=3.315, *p*=0.0106^*^. Non‐significant increase in GM1 levels was observed in the CSF of animals treated with TLN. Unpaired t test: t=0.7122, *p*=0.5082. N=5 rats/group. (E) GM1 levels were measured with LC‐MS in brain tissue 72 h after TLN administration in drinking water (30 mg/kg). TLN‐treated mice displayed increased GM1 levels compared to untreated controls. Unpaired t‐test, t=2.939, *p*=0.0323^*^. N=3 untreated and 4 TLN‐treated wild‐type (*WT)* mice.

Endogenously produced GM1 molecule exerts neurotrophic and neuroprotective properties in the central nervous system (CNS), mainly localized to the outer plasma membrane embedded within lipids. A crucial challenge while targeting CNS availability is the crossing of molecules across the BBB and its bioavailability to specific tissues and cell types. In this study, firstly, we evaluated the biodistribution of free GM1 in CD‐1 rats, which was repeatedly administered via intravenous (i.v.) injections at a dose of 12.3 mg/kg of body weight, followed by plasma collection pretreatment at 4 hours (h) and 8 h after treatment. GM1 levels measured by liquid chromatography‐mass spectrometry (LC‐MS) reached highest concentration within 4 h of treatment and exhibited slow decay kinetics, reaching near basal levels around 24 h after treatment. Further tissue and tissue fluid analysis revealed higher GM1 levels in the brain relative to peripheral tissues, consistent with their endogenous expression in neural tissue, however GM1 levels were almost undetectable within the cerebrospinal fluid (CSF), confirming reduced ability of GM1 to cross the BBB. Moreover, accumulation of GM1 albeit at very low levels was detected within the spleen and liver (Figure 1A).

To enhance the pharmacokinetic properties of GM1, we designed TLN using sphingomyelin and cholesterol as core structural lipids to form a stable bilayer matrix, optimizing both membrane rigidity and biocompatibility. GM1 was functionally integrated into the liposomal membrane to mimic its native localization within neuronal plasma membranes. This incorporation aimed to enhance membrane fusion potential and facilitate interaction with neuronal receptors. The entrapment efficacy is 100% with no residual micellar GM1 detected by HPLC‐SEC. Structural modeling using cryogenic transmission electron microscopy (cryo‐TEM) provided high‐resolution visualization of the TLN architecture and revealed that GM1 is stably embedded within the outer leaflet of the membrane without inducing structural perturbations or compromising vesicle integrity (Figure 1B). Cryo‐TEM further revealed that the liposomes exhibited a predominantly unilamellar morphology with a consistent size distribution, with the majority of the liposomes ranging in size from 20–40 nm, indicating formulation homogeneity and physical stability as measured by the negative Zeta potential of −44.76 mV. Only a minor subpopulation (3.27%) exhibited multilamellar morphology (Figure 1C). The potency of TLN formulation as an Active Pharmaceutical Ingredient (API) is based on the GM1 content 6 mg/ml. The pharmacokinetic profile of TLN was assessed following repeated i.v. administration of 12.3 mg/kg of body weight of nanoliposomal GM1 for four consecutive days in CD‐1 rats, with plasma samples collected at baseline (pre‐treatment), and at 4 and 8 h post‐injection. TLN exhibited pharmacokinetic behavior comparable to that of free GM1, with peak plasma concentrations observed at 4 h, followed by a gradual decline indicative of slow clearance kinetics. Importantly, analysis of brain tissue demonstrated a twofold increase in GM1 levels in animals treated with TLN compared to those receiving free GM1 (Figure 1D), suggesting enhanced CNS penetration and improved BBB permeability of the TLN formulation relative to free GM1. To maximize translational value and given the ease of administration and non‐invasive characteristics of oral formulations, TLN delivery to the brain was evaluated via drinking water over a 72 h period. As measured by LC‐MS, TLN‐treated wild‐type (*WT)* mice exhibited elevated brain GM1 levels relative to untreated controls, demonstrating that oral administration represents a viable approach for TLN delivery (Figure 1E).

### 2.2 TLN Treatment Selectively Acts Upon Mitochondrial, and Synapse Localized Processes

**FIGURE 2:**
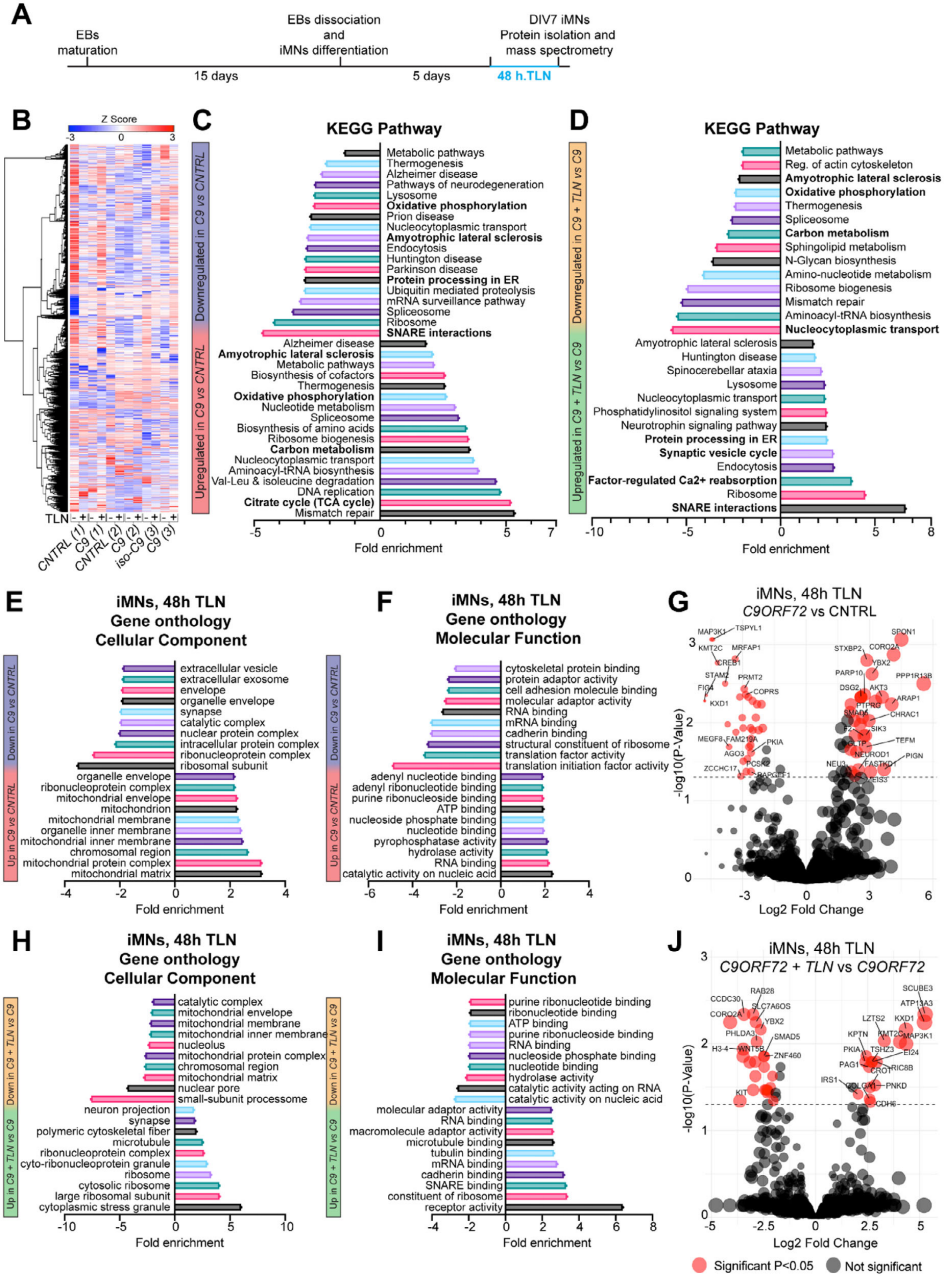
*C9ORF72* iMN proteomic signature and TLN‐induced modulation. (A) Schematic representation of iMNs in culture, protein isolation and mass spectrometry. (B) Heatmap illustrates the pattern of protein expression in controls and *C9ORF72* iMNs with and without TLN treatment. (C–D) KEGG pathway analysis of proteins either up and downregulated in expression in *C9* patients compared to control (*CNTRL)* at baseline (C) and after TLN treatment (D). The expression changes were plotted for proteins showing a change of 1.5 folds and that the change was consistent across all biological replicates. (E–F) Gene Ontology (GO) enrichment analysis of cellular components (E) and molecular functions (F) in *C9ORF72* patient samples compared with *CNTRL* iMNs at baseline. Bars represent fold enrichment for the top GO terms. (G) Volcano plot showing differentially expressed proteins in *C9ORF72* vs *CNTRL* iMNs. Red dots indicate proteins significantly altered with |Log_2_FC| > 1.5 and *p*< 0.05 (adjusted *p* value FDR‐controlled Benjamini and Hochberg multiple test correction). (H–I) GO enrichment analysis of cellular components (H) and molecular functions (I) in *C9ORF72* patient samples before and after TLN treatment. Bars represent fold enrichment for categories altered by TLN. (J) Volcano plot of differentially expressed proteins in *C9ORF72* + TLN vs *C9ORF72*. Red dots indicate proteins significantly changed following TLN treatment with |Log_2_FC| > 1.5 and *p*< 0.05 (adjusted *p* value, FDR‐controlled Benjamini and Hochberg multiple test correction).

We analyzed the signaling pathways and molecular networks altered in disease and the influence of TLN on these processes. To model the most common familial form of ALS; *C9ORF72* and their responses to TLN treatment, we generated induced pluripotent stem cell (iPSC)‐derived motor neurons (iMNs) from 3 *C9ORF72* individual patient lines, two healthy controls and one isogenic ‐*C9ORF72* line. Firstly, we evaluated the cellular uptake of TLN, conjugated with the lipophilic fluorescent probe Dil, in one‐week‐old, induced motor neurons (iMNs) derived from *C9* (3) patient lines and their isogenic controls (See Table 1 in RRIDS for phenotypic description of patient lines). Confocal imaging confirmed robust and efficient internalization of TLN at 24 h across both genotypes, supporting its effective cellular delivery (Figure S1A). Optimal TLN dosage determination was performed on iMNs via Live/Dead assay, establishing 15 µg/mL of TLN as the optimal treatment dose, eliciting consistent and comparable neuroprotective response at 24, 48, and 72 h. (Figure S1B). Based on these data, we performed a mass spectrometric analysis of iMN proteome after 48 h of TLN treatment (see experimental scheme in Figure 2A), which revealed more than 6000 proteins in *Control* and *C9ORF72* iMNs, of which 633 were differentially expressed proteins (DEPs), employing a >1.5‐fold or < 1/1.5‐fold change cutoff (Figure 2B). Among the DEPs, 432 from *C9ORF72* iMNs were upregulated compared to *Control* iMNs, while 201 were downregulated. KEGG pathway analysis, identified statistically overrepresented pathways involved in DNA mismatch repair, TCA cycle, Carbon metabolism and Oxidative phosphorylation (such as RCF proteins and IDH proteins involved in TCA cycle), and nucleocytoplasmic transport (Figure 2C). The downregulated DEPs were associated with SNARE interactions, and Ubiquitin‐mediated proteolysis and protein processing in the ER (Figure 2C), suggestive of altered synaptic vesicle release, and impaired protein folding, handling and degradation. Strikingly, TLN treatment revealed an opposite pattern, whereby a comparison between treated versus untreated *C9ORF72*‐iMNs revealed a down regulation in DNA mismatch repair, TCA cycle, Carbon metabolism and Oxidative phosphorylation pathways and an upregulation in synaptic capacity, SNARE interactions and protein processing in the ER (Figure 2D). Cellular component analysis of DEPs confirmed an enrichment of mitochondrial proteins upregulated within *C9ORF72* iMNs when compared to control iMNs and a significant decrease in expression level of synapse, extracellular vesicle localized proteins and intracellular protein complexes (Figure 2E). Molecular function analyses further confirm the observed profile of DEPs with DNA and mitochondrial functions being upregulated and SNARE functions being downregulated in patient lines (Figure 2F). At an individual protein level, volcano plot confirmed the observed alterations proteins with a significant p value and a |Log_2_FC| < 1.5 such as DNAJC30 involved in mitochondrial process being upregulated while downregulated proteins clustered into synaptic and protein handling (Figure 2G). Cellular component analysis indicated that TLN‐treated *C9ORF72* iMNs show a decline in mitochondria‐associated proteins and an upregulation in proteins associated with synaptic, ribosomal and ribonucleoproteins indicative of reinitiation of gene expression/transcription (Figure 2H). Molecular function further validated an increase in SNARE binding as well as RNA and ribosomal binding and activity (Figure 2I). Individual proteins strongly changed in response to TLN included PNKD (synaptic) and several other proteins activated by neuronal activity such as MAPK3K1, and SMAD5, YBX2 involved in transcription (Figure 2J).

**TABLE 1:** Description and source of the ALS patient and control iPSC lines used in the study. Cell lines were routinely tested for mycoplasma (every 3 months via PCR) and other contaminations (via culture and staining). Frozen stock cell lines were sent out for testing before freezing for future usage. All patient cell lines were obtained from academic source with referenced publications, describing pathological mechanism in ALS.

Subject	Mutation	Age	Sex	Race/nationality	ALSFRS‐R score at screen	Onset till biopsy	Notes
*Control 1*	Healthy	30	M	Asian	N/A	N/A	Made inhouse by Dr. Alexander Laemmle, Institute of Clinical Chemistry and Department of Pediatrics, Inselspital, Bern
*Control 2*	Healthy		M				Commercially available System Bioscience Cat. SC950A‐1 Purchased June 2017
*C9ORF72 (1)*	C9ORF72 positive		M	Caucasian			Donnelly, C.J. et al. Neuron 80 415–428 (2013), Under MTA to Smita Saxena at UBern from Helsinki University Central Hospital, Finland,
*C9ORF72 (2)*	C9ORF72 positive		M	Caucasian			Donnelly, C.J. et al. Neuron 80 415–428 (2013) Under MTA to Smita Saxena UBern from Helsinki University Central Hospital, Finland
*C9ORF72 (3) and iso‐C9ORF72 (3)*	C9ORF72 positive	49	F	N/A			Sareen,D. et al. Sci Transl Med (2013) Cedars Sinai CS52iALS, Under MTA to Smita Saxena Ubern from Cedar Sinai
*C9ORF72 4 and iso‐C9ORF72 (4)*	C9ORF72 positive	38	M	Dutch		1 year	Metha, A.R. et al. Acta Neurophatologica 141, 257–279 (2021) Under MTA to Smita Saxena, from University of Edinburgh

### 2.3 TLN Treatment Selectively Acts on Pathogenic Alterations by Normalizing Protein Expression

**FIGURE 3:**
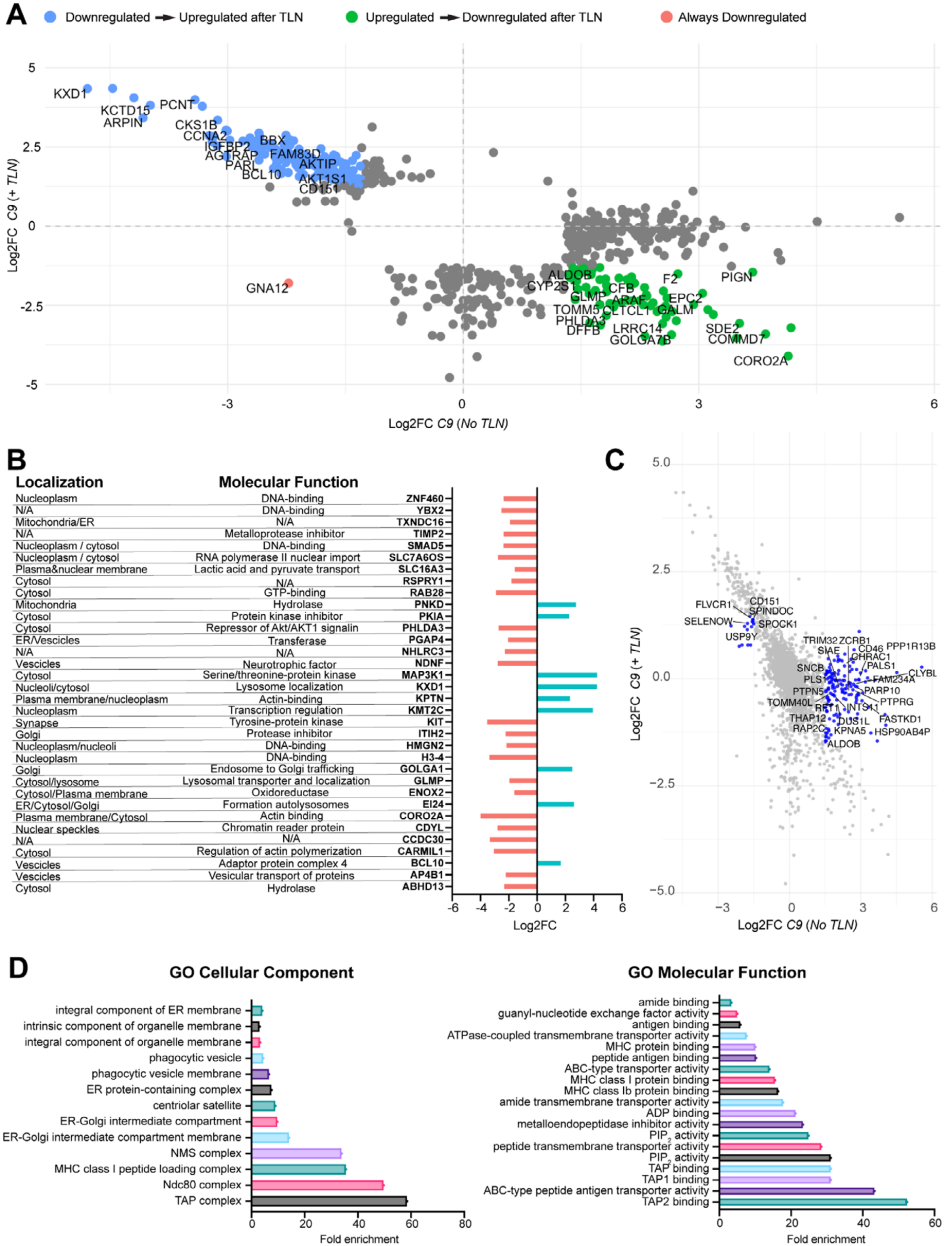
*C9ORF72* iMN pathological signatures are restored upon TLN treatment. (A) Dot plot illustrates proteins that reversed their pattern of expression from being upregulated to being downregulated (green dots) and from reduced expression to being upregulated (blue dots) in *C9ORF72* patient iMNs in response to TLN treatment considering a |Log_2_FC| of ±1.5. (B) Bar plot (right) and table (left) showing localization and molecular function of proteins that present a significant *p* value and a |Log_2_FC| of ± 1.5, whose expression pattern was reversed in *C9ORF72* patient iMNs in response to TLN treatment compared to their baseline. (C) Dot plot highlighted in blue, proteins that were up or downregulated in *C9ORF72* patient iMNs at baseline which returned to normal levels after TLN treatment considering a |Log_2_FC| of ± 1.5. Specifically, 161 proteins upregulated in mutant iMNs were downregulated to below control levels (green dots), while blue dots indicate proteins that transitioned from a downregulated to an upregulated state. (D) GO analysis of cellular components and molecular function of the proteins represented in blue in (C) highlighting normalization of proteins involved in ER‐related processes and inflammation.

Next, we assessed whether TLN harbors neuroprotective potential by activating novel signaling or whether TLN selectively acts upon the pathogenic changes as those changes would occur gradually as a response to cellular stress, toxic protein aggregation and the resultant impairment in cellular homeostasis. To this end, we investigated proteins that reversed their expression profile following TLN treatment and found that approximately 35% of the altered proteome based on the classification of (> 1.5‐fold or < 1.5‐fold change cutoff) in *C9ORF72* iMNs was reversed in expression after TLN treatment, suggesting that TLN treatment reverted the expression of disease‐associated proteins in *C9ORF72* patient iMNs (Figure 3A). Lastly, we analyzed which proteins among the ones that changed their expression levels in response to TLN also present a significant change in *p* value (<0.005). Importantly, internalized TLN acted on proteins localized within multiple cellular organelles and functions, including the ER, mitochondria and synapse suggesting that TLN harbors the capacity to elicit a wide‐ranging pleiotropic effect on iMNs (Figure 3B). We also assessed whether TLN treatment can normalize altered proteome to control conditions, which could identify crucial causal alterations. Around 20% of the altered proteome reverted its expression to control levels (Figure 3C) with a striking portion of those proteins localized within the ER and mainly associated with the ER, ER‐Golgi membranes (Figure 3D). Importantly, one of the strongest effects was observed on the TAP complex, which in neurons is a crucial component of the ER, playing a significant role in the processing and transport of peptides and involved in synaptic stripping in microglia [32, 33] (Figure 3E), indicating that abnormal changes in the ER membrane and function reflect disease‐associated adaptive changes that are normalized in response to TLN's protective effect on pathogenic changes.

### 2.4 TLN Confers Neuroprotection Against Glutamate Toxicity and Restores Mitochondrial Function in Patient‐Derived iMNs

**FIGURE 4:**
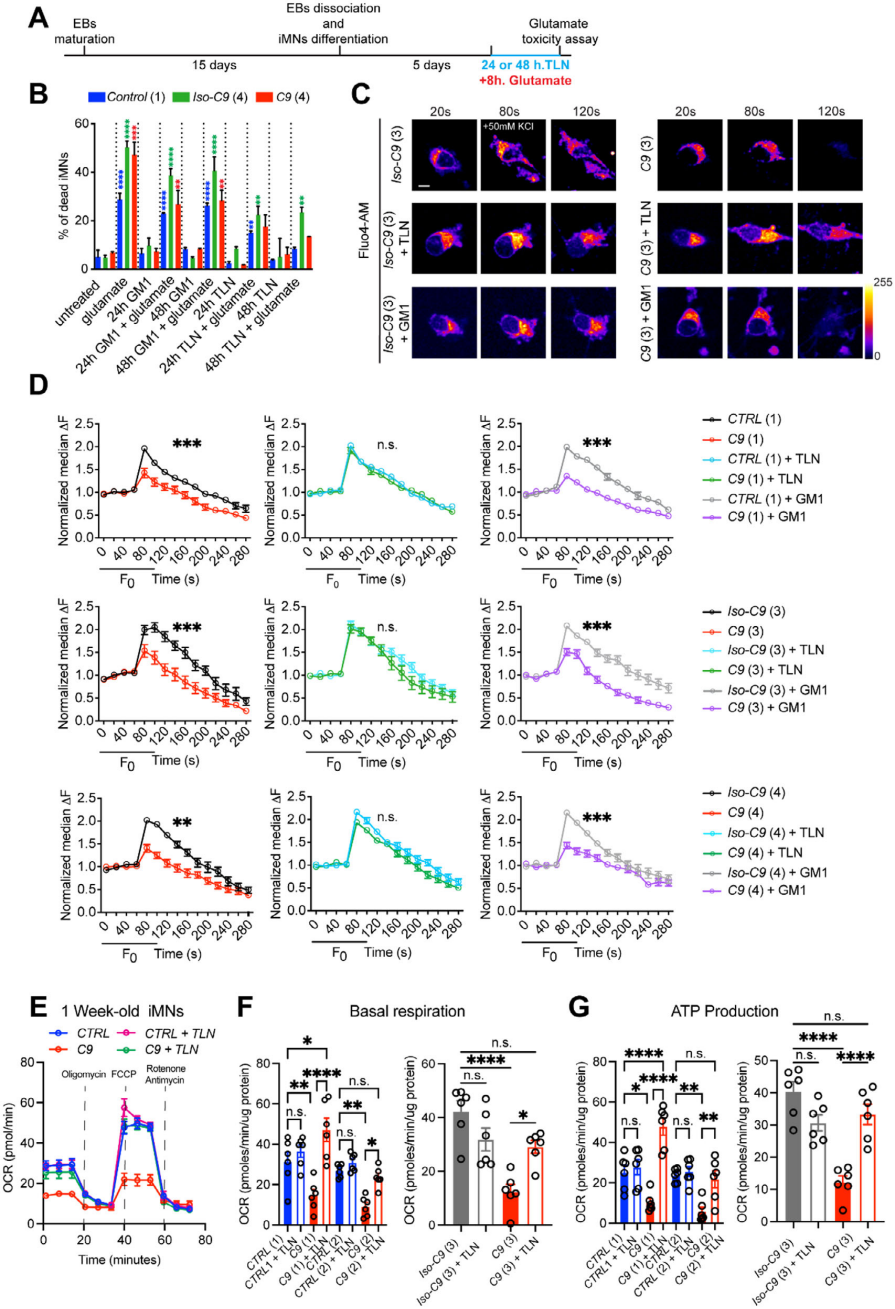
TLN rescues mitochondrial calcium uptake and mitochondria functionality in *C9ORF72* iMNs. (A) Experimental timeline for glutamate toxicity assay in iMNs. (B) Bar graph illustrates the percentage of dead iMNs cultured in the presence of 15 µg/ml of TLN or 15 µg/ml of free GM1 for 24 and 48 h. Response to glutamate toxicity was assessed via 8 h of 10 µM glutamate administration to these cultures. 24 h of TLN treatment is sufficient to display a neuroprotective effect on iMNs, and the neuroprotective response was maintained at 48 h of TLN treatment, especially in mutant *C9* (4) iMNs. One‐way ANOVA was performed (F = 45.84), followed by Dunnett's multiple comparisons test. Statistical significance is indicated in the figure: blue asterisks indicate comparisons versus control (*CTRL* (1))‐untreated, green asterisks indicate comparisons versus *Iso‐C9* (4)‐untreated, and red asterisks indicate comparisons versus *C9* (4)‐untreated. (C) Representative images of mitochondria calcium imaging in *Control* and *C9* patient with and without TLN treatment at baseline and after stimulation with 50 mM KCl. Scale bar: 20 µm. (D) Calcium imaging traces of different *Control* and *Isogenic control* lines with corresponding *C9* patients’ 1‐week‐old iMNs lines. *Note*: TLN restores mitochondrial calcium transients compared to GM1 alone. Multiple t test at 100s: *CTRL* (1) mean: 1.639; *C9* (1) mean: 1.224, *p* value: 0.004; *C9* (1) mean: 1.224; *C9* (1) + GM1 mean: 1.204, *p* value: 0.85; *C9* (1) mean: 1.224; *C9* (1) + TLN mean: 1.666, *p* value: 0.0006; *Iso‐C9* (3) mean: 1.923; *C9* (3) mean: 1.250, *p* value <0.0001; *C9* (3) mean: 1.250; *C9* (3) + GM1 mean: 1.312, *p* value: 0.55; *C9* (3) mean: 1.250; *C9* (3) + TLN mean:1.761, *p* value:<0.0001; *Iso‐C9* (4) mean: 2.046; *C9* (4) mean: 1.384, *p* value: 0.001; *C9* (4) mean: 1.384; *C9* (4) + GM1 mean: 1.461, *p* value:0.66; *C9* (4) mean: 1.384; *C9* (4) + TLN mean: 1.948, *p* value: 0.002. (E) Seahorse representative traces of mitochondrial oxygen consumption rate (OCR). (F) Bar plots of seahorse basal respiration quantification. One‐way ANOVA: *CTRL* (1&2) and *C9* (1&2): F=13.23, *p* value <0.0001. Šidák's multiple comparisons: *CTRL* (1) vs *C9* (1) ^**^; *CTRL* (1) vs *CTRL* (1) + TLN n.s. *C9* (1) vs *C9* (1) + TLN ^****^; *CTRL* (2) vs *C9* (2) ^**^; *CTRL* (2) *vs CTRL* (2) + TLN n.s. *C9* (2) vs C9 (2) + TLN ^*^. One‐way ANOVA: *Iso‐C9* (3) and *C9* (3): F=13.01, *p* value <0.0001. *Iso‐C9* (3) vs *C9* (3)^****^; *Iso‐C9* (3) vs *Iso‐C9* (3) + TLN n.s.; *C9* (3) vs *C9* (3) + TLN ^*^. (G) Bar plots of seahorse ATP production quantification. One‐way ANOVA: *CTRL* (1&2) and *C9* (1&2): F=16.06, *p* value <0.0001. Šidák's multiple comparisons: *CTRL* (1) vs *C9* (1) ^*^; *CTRL* (1) vs *CTRL* (1) + TLN n.s. *C9* (1) vs *C9* (1) + TLN ^****^; *CTRL* (2) vs *C9* (2) ^**^; *CTRL* (2) vs *CTRL* (2) + TLN n.s. *C9* (2) vs *C9* (2) + TLN ^**^. One‐way ANOVA: *Iso‐C9* (3) and *C9* (3): F=19.32, *p* value <0.0001. *Iso‐C9* (3) vs *C9* (3)^****^; *Iso‐C9* (3) vs *Iso‐C9* (3) + TLN n.s.; *C9* (3) vs *C9* (3) + TLN ^****^.

*C9ORF72* motor neurons show enhanced susceptibility to stressors such as nutrient depletion or excess glutamate. Thus, to evaluate the neuroprotective potential of TLN on *C9ORF72* iMNs, glutamate toxicity assay was performed. The experimental plan was based on our previous observation that a single administration of TLN at 15 µg/kg to iMNs was sufficient to elicit a strong and comparable neuroprotective response at 24 and 48 h (Figure S1B). Therefore, one‐week‐old iMNs were cultured in the presence of TLN or free GM1 for either 24 or 48 h. During this TLN treatment period, iMNs were exposed to 10 µM glutamate for 8 h, followed by the quantification of surviving neurons using a Live/Dead assay (Figure 4A). TLN treatment for 24 h was sufficient to markedly attenuate glutamate‐induced cytotoxicity in *C9ORF72* iMNs, whereas GM1 alone failed to confer protection, resulting in continued neuronal loss. Notably, 48 h of TLN treatment significantly protected iMNs as compared to GM1 alone highlighting TLN's superior neuroprotective efficacy (Figure 4B). Given that a large proportion of DEPs in *C9ORF72* iMNs were mitochondrial, and previous studies have shown dysregulation of mitochondrial calcium (Ca^2^⁺) uptake, we assessed mitochondrial Ca^2^⁺ dynamics in iMNs. Live‐cell imaging revealed that *C9ORF72* iMNs exhibited abnormally elevated baseline mitochondrial Ca^2^⁺ and a markedly attenuated response to KCl‐induced depolarization, indicative of impaired mitochondrial Ca^2^⁺ handling (Figure 4C). TLN treatment effectively reversed these deficits, normalizing both basal Ca^2^⁺ levels and stimulus‐induced transients in multiple patient iMNs (*p* < 0.01), whereas GM1 alone failed to produce any significant rescue (Figure 4D). Nanoliposomes lacking GM1, referred to as base vesicles (BV) exhibited no restorative effect on mitochondrial Ca^2^⁺ uptake, indicating that the functional rescue relied on GM1‐loaded TLN and was not due to the liposomal formulation alone (Figure S1C). As deficits in mitochondrial Ca^2^⁺ uptake correlate with functional deficits, we examined overall mitochondrial function. Mitochondrial respiration, assessed via Seahorse extracellular flux analysis, revealed a profound impairment in basal oxygen consumption rate (OCR) in *C9ORF72* iMNs, reflecting a bioenergetic deficit characteristic of ALS‐associated mitochondrial dysfunction (Figure 4E). TLN treatment partially restored mitochondrial respiratory capacity, highlighting its potential to rescue mitochondrial deficits linked to *C9ORF72* pathology (Figure 4F). ATP production was markedly impaired in *C9ORF72* iMNs, consistent with compromised mitochondrial bioenergetics underlying ALS pathology. TLN treatment robustly restored ATP production, effectively rescuing metabolic function to near‐physiological levels. In contrast, GM1 treatment failed to produce a significant effect. These findings underscore TLN's therapeutic efficacy in re‐establishing mitochondrial Ca^2^⁺ homeostasis and ATP synthesis, two core intrinsic deficits driving motor neuron vulnerability in *C9ORF72*‐linked ALS (Figure 4G).

### 2.5 Efficient Uptake and Accumulation of TLN in the Brain and Spinal Cord

**FIGURE 5:**
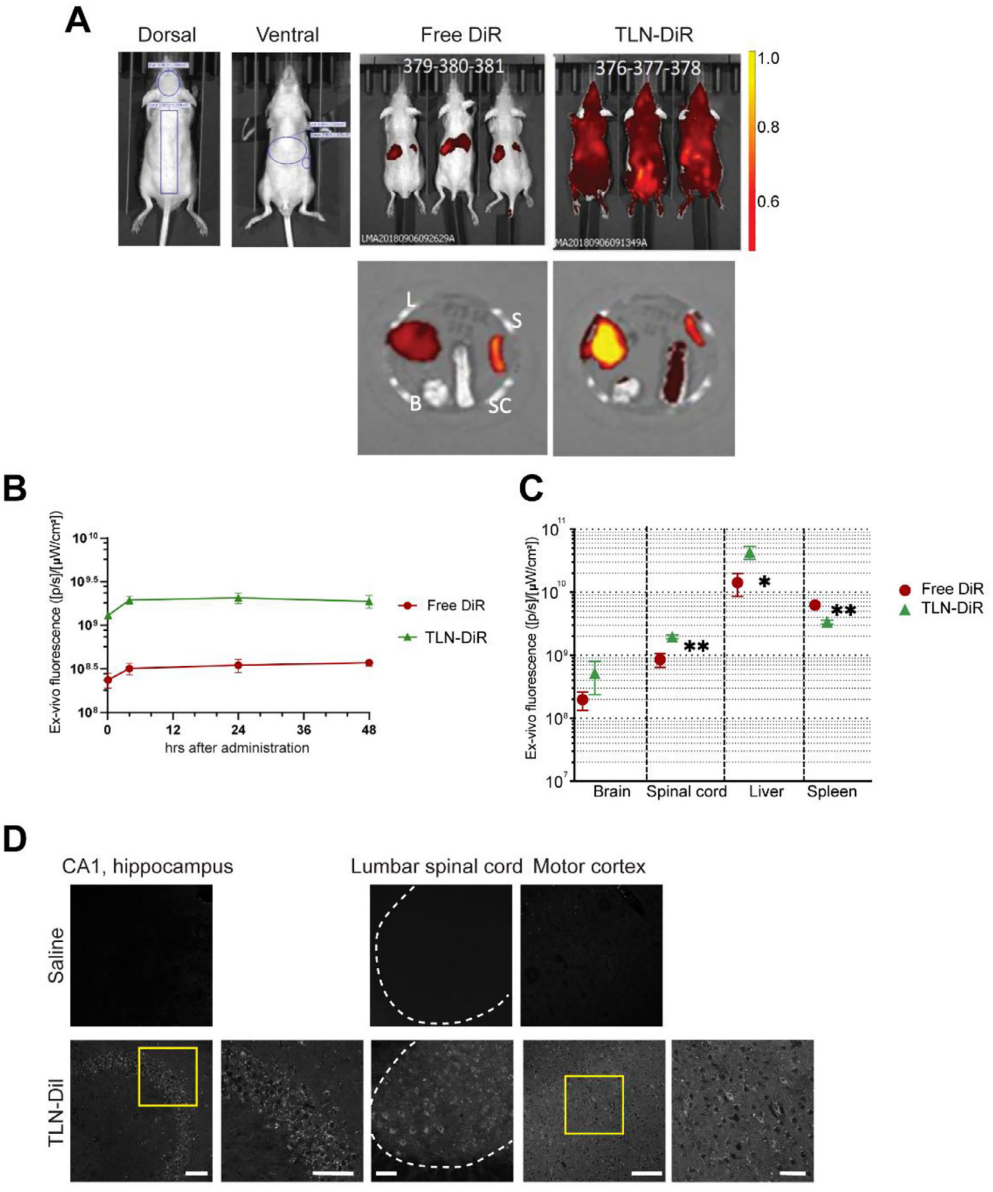
In vivo assessment of TLN biodistribution in mice. (A) Segmentation example of Regions of interest (ROI) placed on the dorsal (left) and ventral (right) view. Representative images of dorsal in vivo fluorescence signal in whole mice at 48 h (Right). N=3 mice/group (B) Quantification of individual fluorescence signal in the brain, measured at 5 min, 4 h, 24 and 48 h post single i.v. administration of TNL‐DiR (15 mg/kg) and free DiR (mean ± SD, N=3, background subtracted). (C) Comparative Quantification of individual fluorescence signal accumulated in the brain (B), spinal cord (SC), liver (L) and spleen (S) 48 h post i.v. administration (mean ± SD, N=3 images, background subtracted). Unpaired t test TLN vs Free‐DiR: spinal cord t = 7.762, *p* = 0.0015^**^, liver t = 4.361, *p* = 0.0121^*^; spleen t = 6.877, *p* = 0.0023^**^. (D) The biodistribution of TLN‐Dil in the CNS measured after 24 h of post i.v. injection at a dose of 15 mg/kg. Dil positive neurons were detected in CA1 hippocampus in the Layer V of the primary motor cortex and in the ventral horn of lumbar spinal cord within large neurons. Scale bar: hippocampus 200 & 100 µm; spinal cord 200 µm; M1 250 & 50 µm. N=3 animals/group.

We next assessed the therapeutic potential of TLN using in vivo in preclinical rodent models of ALS. To quantitatively assess the in vivo biodistribution and target tissue accumulation of TLN, we employed non‐invasive tomographic imaging. TLN was conjugated to the near‐infrared fluorescent dye DiR and administered i.v. to adult mice (*n* = 3 per group (DiR only, and TLN‐DiR, (15 mg/kg of body weight), followed by longitudinal imaging at 12 h intervals over a 48 h period. This approach enabled dynamic tracking of formulation kinetics and tissue‐specific uptake. The liver showed the highest intensity of fluorescence, followed by the spleen, however DiR labeling was not detected within the brain or spinal cord consistent with its inability to cross the BBB (Figure 5A). TLN‐DiR had higher and consistent levels of accumulated fluorescence over a 48 h period compared to free‐DiR (Figure 5B). We also quantified the TLN‐DiR organ biodistribution for four organs: liver, spleen, brain and spinal cord, revealing significant accumulation of TLN‐DiR within the brain and the spinal cord (Figure 5C). To investigate the regional accumulation of TLN within the central nervous system at the cellular level, mice were administered TLN‐Dil via i.v. injection, while control animals received saline. Brain and spinal cord tissues were collected for 48 h post injection for histological analysis. Confocal imaging revealed robust Dil fluorescence in TLN‐treated animals, indicating efficient neuronal uptake. Notably, Dil‐positive neurons were prominently localized in the CA1 region of the hippocampus, cortical Layer V of the primary motor cortex, and lumbar spinal cord, primarily within the large motor neurons in the ventral horn (Figure 5D). These data indicate that TLN can cross the BBB and target key neuroanatomical regions relevant to ALS pathology.

### 2.6 TLN Administration Ameliorates Motor Deficits and Extends Survival in Familial ALS Mouse Models

**FIGURE 6:**
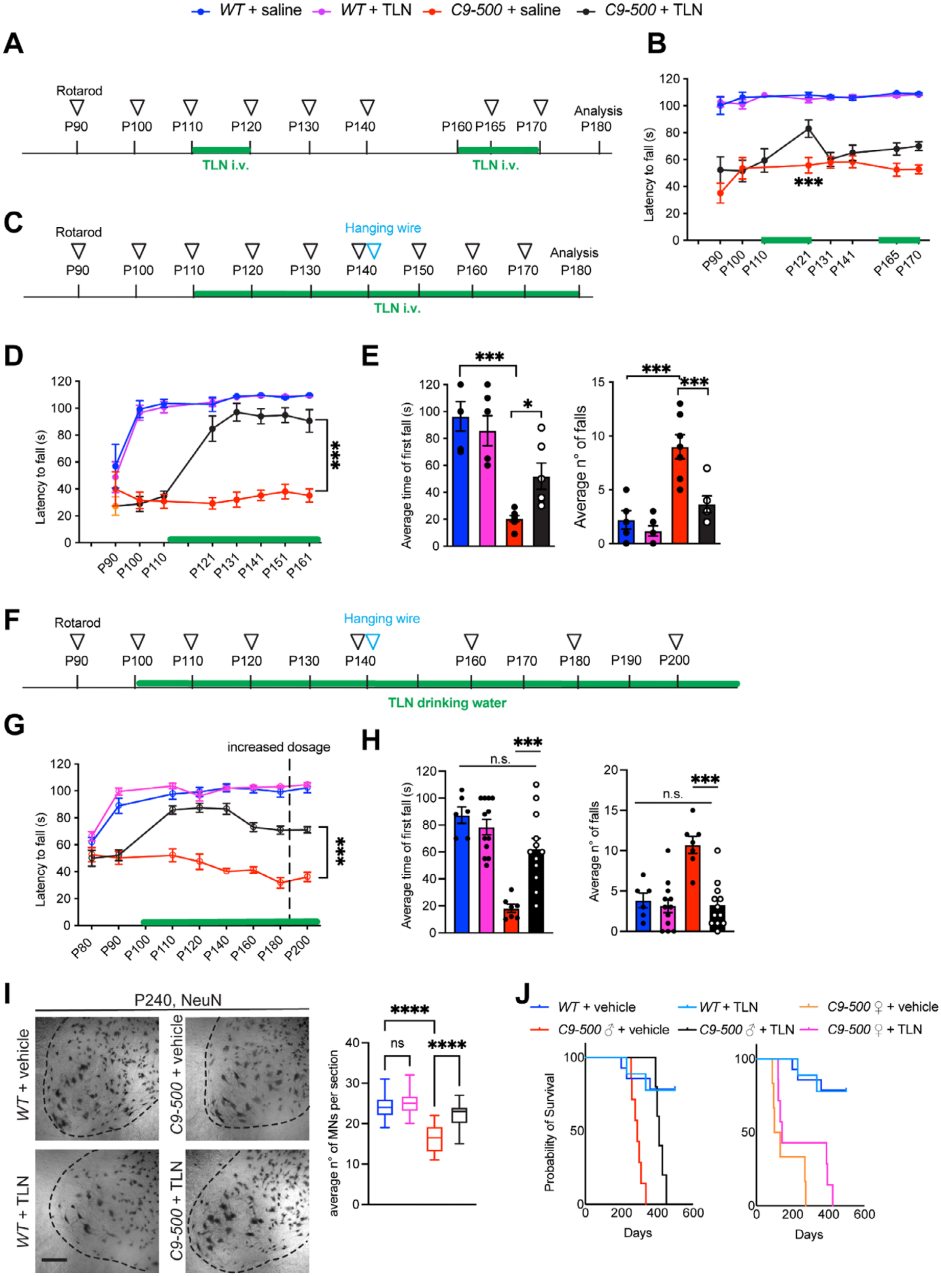
TLN treatment improves motor function and increases survival in two ALS mouse models. (A) Experimental timeline for discontinuous TLN i.v. administration. TLN was administered between P110 to P120 and P160 to P170 at a dose of 15 mg/kg/2 days. (B) Inverse rotarod test displayed as latency to fall. Ten days of i.v. TLN treatment between P110 and P120 is sufficient to ameliorate motor impairments in *C9‐500* animals (Two‐way ANOVA interaction F_(21,160)_= 1.341, *p* value= 0.16, time F_(7,160)_= 4.099, *p* value= 0.0004, genotype F_(3,160)_= 271,5, *p* value <0.0001. Bonferroni correction for multiple comparisons: P110 *C9‐500* vs *C9‐500* + TLN n.s.; P121 *C9‐500* vs *C9‐500* + TLN ^***^). N=6 *WT* + saline, 6 *C9‐500* + saline, 6 *WT* + TLN, 7 *C9‐500* + TLN. Scale bar: 200µm. (C) Experimental timeline of continuous TLN i.v. administration. at a dosage of 15 mg/kg/2 days. (D) Inverse rotarod test displayed as latency to fall. i.v. administration of TLN from P110 can rescue motor coordination in *C9‐500* animals (Two‐way ANOVA interaction F_(21, 72)_= 4.539, *p* value <0.0001, time F_(7,72)_= 5.462, *p* value <0.0001, genotype F_(3,72)_= 132.7, *p* value <0.0001. Bonferroni correction for multiple comparisons: P110 *C9‐500* + saline vs *C9‐500* + TLN n.s.; P121 to P161 *C9‐500* + saline vs *C9‐500* + TLN ^***^). N=5 *WT* + saline, 7 *C9‐500* + saline, 6 *WT* + TLN, 6 *C9‐500* + TLN. (E) Hanging wire test illustrated as bar graph. TLN treatment restores muscle strength in *C9‐500* animals compared to saline controls. Left: average time of first fall, one‐way ANOVA: F=15.90, *p* value <0.0001; Šidák's multiple comparisons: *WT* + saline vs *C9‐500* + saline ^***^, *C9‐500* + saline vs *C9‐500* + TLN ^*^. Right: average no. of falls, one‐way ANOVA: F=17.44, *p* value <0.0001; Šidák's post hoc test for multiple comparisons: *WT* + saline vs *C9‐500* + saline ^***^, *C9‐500* + saline vs *C9‐500* + TLN ^***^. N=5 *WT* + saline, 7 *C9‐500* + saline, 6 *WT* + TLN, 6 *C9‐500* + TLN. (F) Experimental timeline for the administration of TLN in drinking water. TLN was administered continuously in drinking water from P100 to P180 at a dose of 7.5 mg/kg and later doubled to 15 mg/kg until euthanasia, to account for the strong symptomatic stage of the disease. Control mice were given water with stevia termed as vehicle throughout the manuscript. (G) Inverse rotarod test plotted as latency to fall. Drinking water administration of TLN from P100 onward rescues motor performance in treated *C9‐500* animals. Two‐way ANOVA interaction F_(21,263)_= 9.041, *p* value <0.0001, time F_(7,263)_= 23.03, *p* value <0.0001, genotype F_(3,263)_= 340.0, *p* value <0.0001. Bonferroni correction for multiple comparisons: P80‐P90 *C9‐500* + vehicle vs *C9‐500* + TLN n.s.; P110 to P200 *C9‐500* + vehicle vs *C9‐500* + TLN ^***^). N=6 *WT* + vehicle, 11 *C9‐500* + vehicle, 12 *WT* + TLN, 12 *C9‐500* + TLN. (H) Hanging wire test illustrated as bar graph. TLN treatment in drinking water is able to restore muscle strength in *C9‐500* animals compared to vehicle controls. Left: average time of first fall, one‐way ANOVA: F=16.41, *p* value <0.0001; Šidák's post hoc test for multiple comparisons: *WT* + vehicle vs *C9‐500* + vehicle ^***^, *C9‐500* + vehicle vs *C9‐500* + TLN ^***^. Right: average no. of falls, one‐way ANOVA: F=13.46, *p* value <0.0001; Šidák's multiple comparisons: *WT* + vehicle vs *C9‐500* + vehicle ^***^, *C9‐500* + vehicle vs *C9‐500* + TLN ^***^. N=6 *WT* + vehicle, 7 *C9‐500* + vehicle, 12 *WT* + TLN and 12 *C9‐500* + TLN. (I) Representative images of the ventral horn of the lumbar spinal cord of *WT* and *C9‐500* + vehicle and treated with TLN in drinking water. Quantitative analysis shows a significant decrease in the average number of MNs in *C9‐500* + vehicle animals compared to animals treated with TLN (One‐way ANOVA: F=38.29, *p* value <0.0001; Šidák's post hoc test for multiple comparisons: *WT* + vehicle vs *C9‐500* + vehicle ^****^, *C9‐500* + vehicle vs *C9‐500* + TLN ^****^). (J) Survival curves of the different genotypes and treated groups. Log‐rank test: chi square: 94.39, *p* value <0.0001. Median survival *C9‐500* + vehicle males: 290 days, *C9‐500* + TLN males: 411 days, *C9‐500* + vehicle acute phenotype females: 98 days, *C9‐500* + TLN acute phenotype‐females: 133 days, *C9‐500* + vehicle slow‐progressing females: 275 days, *C9‐500* + TLN slow‐progressing females: 390 days. N=14 *WT* + vehicle, 13 *C9‐500* + vehicle, 9 *WT* + TLN, 12 *C9‐500* + TLN.

To evaluate the therapeutic efficacy of TLN in vivo, we employed both acute and chronic administration strategies across multiple behavioral and histological assays in the *C9‐500* mouse model of ALS. These mice display RNA foci, pTDP43 aggregates and robust accumulation of DPRs, all of which are pathological hallmarks of *C9ORF72*‐ALS/FTD [34, 74]. To mimic the clinical setting in which patients present with symptoms, we initiated acute i.v. administration of TLN (15 mg/kg/2 days) between postnatal days (P)110–P120 (Figure 6A), a stage at which *C9‐500* mice exhibit pronounced cellular stress pathology [10]. Although substantial differences were observed at baseline rotarod measurements between *WT* and *C9‐500* mice, TLN‐treated *C9‐500* mice displayed enhanced motor performance by P121 compared to untreated *C9‐500* littermates. Halting the treatment reversed the acute beneficial effect, however restarting TLN treatment forty days later, at an age when mice exhibit stronger disease symptoms, still elicited beneficial effects (Figure 6B). Building further on this observation, we next initiated chronic i.v. TLN delivery from P110 (Figure 6C), which rescued rotarod performance over an extended time course (P110–P161), with TLN‐treated *C9‐500* animals exhibiting significantly prolonged latency to fall (Figure 6D). During this chronic treatment regime, we also performed a hanging wire test, TLN restored muscle strength in *C9‐500* mice, as reflected by both an increased time to first fall and a reduced number of falls (Figure 6E). As oral formulations enable non‐invasive, patient‐friendly administration, which significantly improves compliance, especially for long‐term treatments, we chronically administered TLN (7.5 mg/kg/dose/day from P100‐P185 and doubled the dose to 15 mg/kg/day from P185‐end stage) in drinking water (Figure 6F). Oral administration of TLN also significantly and consistently improved motor function in *C9‐500* mice beginning at P110 (Figure 6G). This was further confirmed by hanging wire performance, with TLN‐treated *C9‐500* animals showing substantial improvements in muscle strength (Figure 6H). While TLN treatment did not affect body weight which remained unchanged and consistent between both genotypes and conditions (Figure S2A), TLN treatment prevented *C9‐500* mice from progressing to a severe clasping phenotype (Figure S2B). We further evaluated muscle pathology by Hematoxylin and eosin (H&E) staining, which revealed pathological features characteristic of muscle denervation, including angular fibers and centrally located nuclei, in *C9‐500* mice treated with vehicle. These features were absent from TLN‐treated *C9‐500* muscles. Nicotinamide adenine dinucleotide (NADH) staining demonstrated a preserved checkerboard pattern of oxidative fiber distribution in *WT* muscle, which was markedly disrupted in saline‐treated *C9‐500* mice. In contrast, TLN treatment restored and maintained this fiber‐type pattern, indicating preserved muscle architecture and mitochondrial function (Figure S2C). Immunofluorescence labeling of lumbar spinal cord demonstrated a significant preservation of large ventral horn neurons in TLN‐treated *C9‐500* mice relative to vehicle‐treated counterparts, indicative of robust neuroprotective effects (Figure 6I). Importantly, this cellular preservation translated into a marked survival benefit since TLN treatment significantly extended the lifespan of both male and female *C9‐500* mice, irrespective of disease progression rate. Median survival increased by more than 121 days for males, 115 days for slow progressive females and 35 days for acutely progressing females (Figure 6J), underscoring the therapeutic potential of TLN in modifying disease course and delaying mortality in ALS.

Next, we longitudinally examined whether long‐term oral TLN administration elicits systemic toxicity. To this end, we monitored body weight, hydration status, and serum biochemical markers in late‐symptomatic *C9‐500* mice which had received 15 mg/kg of TLN in drinking water from P200 to P255: Figure S3A). As observed previously in Figure S2A, long term TLN administration at high doses did not alter body weight in either *WT* or *C9‐500* symptomatic animals at any measured time point (Figure S3B). Similarly, hydration ratios measured via EchoMRI remained stable across both groups, showing no signs of dehydration or fluid imbalance during treatment (Figure S3C). Liver and kidney function were determined via serum alanine aminotransferase (ALT), aspartate aminotransferase (AST) liver enzymes, and urea levels in *C9‐500* mice and *WT* littermates at P200 (pre‐treatment) and after their prolonged treatment with TLN in drinking water. Untreated P200, *C9‐500* mice showed a mild baseline increase in ALT and AST compared to untreated *WT* controls. However, TLN treatment did not further exacerbate ALT or AST levels in those mice. (Figure S3D). Serum urea concentrations were similarly unaffected by TLN administration, with no differences observed between *WT* and *C9‐500* mice (Figure S3E). As abnormally high levels of circulating pro‐inflammatory cytokine IL‐1beta (IL‐1β) are observed in ALS patients, and correlates with disease severity [35], we found that pre‐treatment *C9‐500* mice displayed higher serum IL‐1β levels, which were not exacerbated after TLN treatment. Together, these data demonstrate that chronic TLN therapy is well tolerated and does not induce or worsen organ function or immune responses.

As *SOD1‐G93A, TDP‐43* and *C9ORF72* models reflect distinct disease mechanisms; yet share conserved pathological alteration, we tested TLN in a second familial ALS mouse model. To this end, we chronically administered i.v. TLN to *SOD1‐G93A* mice from P110, corresponding to a symptomatic stage (Figure S4A). TLN treatment at 15 mg/kg/2 days significantly improved motor coordination in *SOD1‐G93A* mice, as measured by the rotarod performance (Figure S4B). One cardinal hallmark of ALS pathology in *SOD1‐G93A* mice is gradual weight loss, TLN‐treated *SOD1‐G93A* mice maintained body weight through disease progression, in contrast to the pronounced weight loss observed in saline‐treated counterparts by P150 (Figure S4C). TLN administration markedly mitigated the hindlimb clasping phenotype, a hallmark of neurodegeneration in *SOD1‐G93A* mice, further supporting its therapeutic benefit in familial ALS preclinical models (Figure S4D).

### 2.7 TLN Treatment Restores Mitochondrial and Synaptic Function and Ameliorates Inflammation in C9‐500 ALS Mice

**FIGURE 7:**
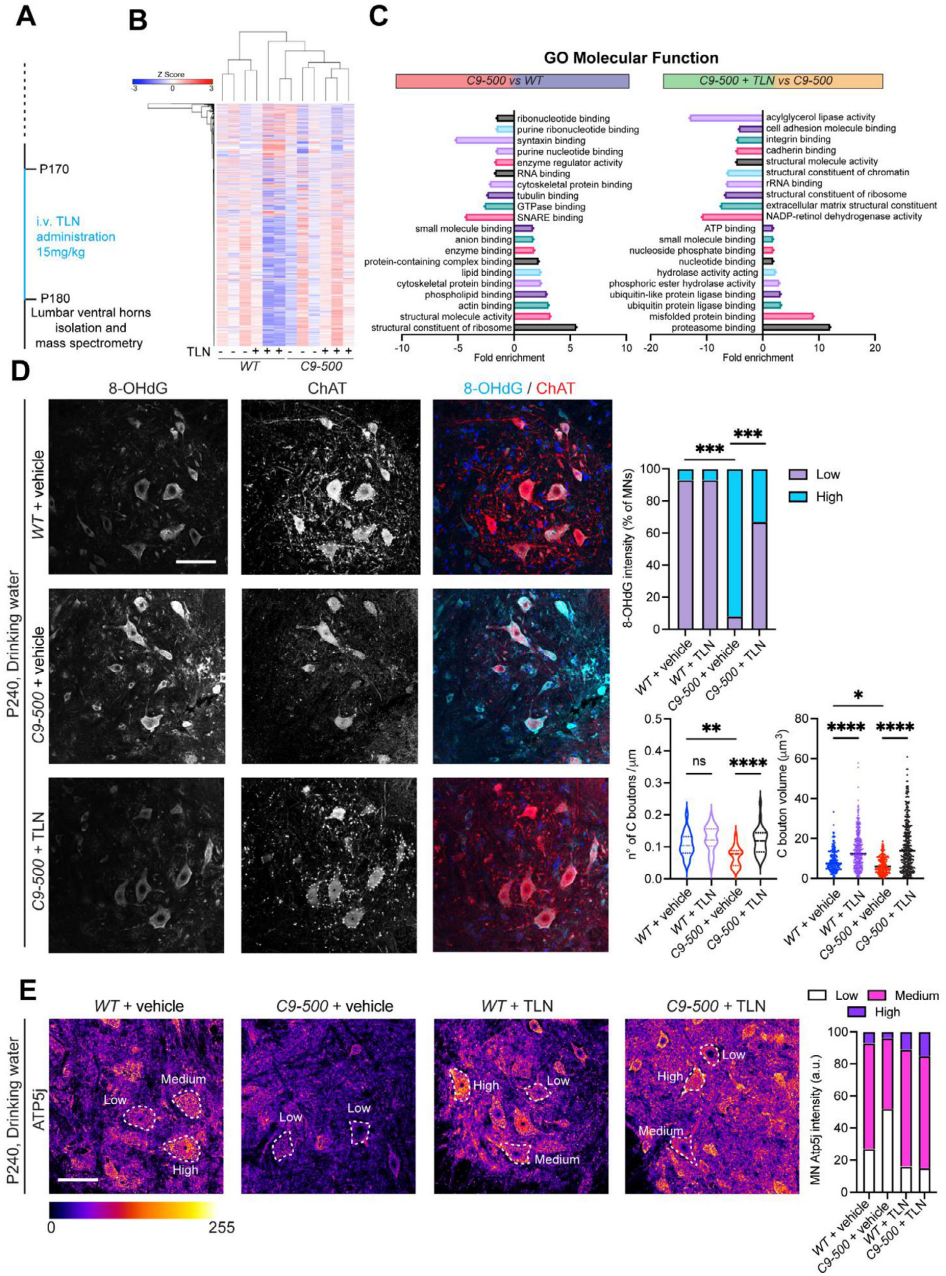
TLN treatment sustains cholinergic synaptic inputs and mitochondria functionality in vivo. (A) Experimental timeline of proteomic analyses of i.v. treated symptomatic *C9‐500* and *WT* mice. TLN or saline was administered between P170 to P180 at a dose of 15 mg/kg every alternate day and ventral horns of the lumbar region of the spinal cord was isolated for mass spectrometry. (B) Heatmap illustrates the overall pattern of protein expression of *WT* and *C9‐500* spinal ventral horn ‐/+ TLN treatment. (C) Bar plot illustrating gene ontology (GO) for molecular function of *C9‐500* animals vs *WT* and after TLN administration. (D) Representative immunofluorescence staining for 8‐OHdG (DNA/RNA damage marker) and ChAT. Quantitative analyses (Q.A.) of 8‐OHdG intensity revealed a reduction in oxidative stress and preservation of cholinergic inputs onto *C9‐500* spinal motor neurons treated with TLN in drinking water (8‐OHdG *WT* + vehicle vs *C9‐500* + vehicle Chi‐square: 144.5, *p*<0.0001; 8‐OHdG *C9‐500* + vehicle vs *C9‐500* + TLN Chi‐square: 74.26, P<0.0001; one‐way ANOVA; C boutons number: F=12.55, *p*< 0.0001, Šidák's multiple comparisons: *WT* + vehicle vs *C9‐500* + vehicle *p*=0.0024, *C9‐500* + vehicle vs *C9‐500* + TLN, *p*<0.0001; one‐way ANOVA; C boutons volume: F=64.55, *p* value< 0.0001, Šidák's multiple comparisons: *WT* + vehicle vs *C9‐500* + vehicle P=0.0388, *C9‐500* + vehicle vs *C9‐500* + TLN, *p*<0.0001). N=3 animals/genotype/treatment. Scale bar: 100µm. (E) Representative confocal images of the mitochondrial protein Atp5j. Average intensity was divided in 3 different categories of intensity, Q.A. display a dramatic decrease in Atp5j levels in *C9‐500* + vehicle motor neurons whereas Atp5j expression was preserved and comparable to *WT* + vehicle in TLN‐treated mutant animals (Chi‐square *WT* + vehicle vs *C9‐500* + vehicle: 15.96, *p*=0.0006; *C9‐500* + vehicle vs *C9‐500* + TLN Chi‐square: 31.96, *p*<0.0001). Scale bar: 50µm.

To investigate the molecular basis of TLN's neuroprotective effects in *C9‐500* ALS mice, we conducted comprehensive mass spectrometry‐based proteomic profiling of lumbar spinal cord ventral horns following i.v. TLN administration. We administered TLN for 10 days in highly symptomatic *C9‐500* mice from P170‐P180 (Figure 7A). This unbiased approach revealed pronounced alterations in protein expression between *C9‐500* and *WT* controls, particularly involving pathways associated with mitochondrial dysfunction, synaptic maintenance (SNARE binding, Synataxin‐1 binding), and oxidative stress response (Lipid and phospho‐lipid binding), as previously observed in human *C9ORF72* iMNs (Figures 7B,C and (Figure S5A,B). Specifically, several processes associated with protein handling, proteasome function and ubiquitin binding were upregulated indicative of improved protein handling capacity after TLN treatment (Figure 7C). Notably, a normalization of DEPs to *WT* levels was observed in the ventral lumbar spinal cords of *C9‐500* mice following TLN treatment, consistent with the proteomic normalization observed in TLN‐treated human iMNs. A dot plot visualization revealed that among the more than 300 DEPs, significantly upregulated or downregulated at baseline in *C9‐500* ventral horn, approximately 30 % returned to expression levels comparable to *control* upon TLN administration (Figure S5C). These proteins spanned key functional categories implicated in ALS pathology. Gene Ontology (GO) enrichment analysis of this normalized subset highlighted recovery in molecular functions and cellular components essential to neuronal health, including those involved in autophagy, synaptic vesicle cycling, and mitochondrial maintenance. Specifically, TLN restored the expression of proteins involved in autophagosome formation and lysosomal trafficking, processes known to be disrupted in *C9ORF72*‐linked ALS due to defects in vesicle clearance and protein turnover. Importantly, synaptic metabolism‐related proteins, which were downregulated in untreated *C9‐500* mice, were significantly upregulated with TLN treatment, indicating improved synaptic resilience and neurotransmission capacity (Figure S5D). This convergence of proteomic profiles across species and models underscores the robustness and translational relevance of TLN's molecular effects. Complementary immunofluorescence studies provided spatial and cellular resolution of these molecular changes, demonstrating that TLN markedly reduced oxidative damage within motor neurons, as indicated by decreased 8‐hydroxyguanosine (8‐OHdG) staining, a sensitive marker of DNA/RNA oxidative lesions (Figure 7D). Concurrently, TLN preserved the density and volume of cholinergic synaptic boutons (C‐boutons) on motor neurons, reflecting sustained neuromuscular connectivity and synaptic input critical for motor neuron function (Figure 7D). Moreover, confocal imaging of the mitochondrial marker Atp5j revealed a significant preservation of mitochondrial ATP synthase expression in TLN‐treated *C9‐500* motor neurons, implicating enhanced mitochondrial bioenergetic capacity as a mechanism underlying functional improvements (Figure 7E).

We next investigated whether the reduction in oxidative stress was also accompanied by decreased inflammatory signaling and a normalization of microglial reactivity in response to TLN treatment. Proteomic analysis of the lumbar spinal cord revealed dysregulation of several proteins involved in inflammation and cellular stress responses in *C9‐500* mice in both pre‐symptomatic stages and symptomatic stages (Figure S6A), indicating that inflammatory processes are already present before overt symptoms onset. To define the cellular correlates of these proteomic alterations, we examined microglial morphology in the ventral horn. *C9‐500* vehicle‐treated mice showed reduced Iba1⁺ microglial ramification compared to *WT* controls, as indicated by fewer Sholl intersections across radial distances from the soma. TLN treatment significantly increased microglial branching in *C9‐500* mice, restoring morphological complexity to *WT* state (Figure S6B). Furthermore, we examined astrocytic reactivity using GFAP staining. *C9‐500* vehicle‐treated mice displayed a pronounced increase in GFAP⁺ immunoreactivity within the spinal ventral horn, consistent with astrogliosis. TLN treatment markedly reduced the GFAP⁺ area in *C9‐500* mice, while having no effect on *WT* animals (Figure S6C). Finally, analysis of circulating pro‐inflammatory cytokines indicated that serum levels of IL‐1β, IL‐6, and TGF‐β1 were elevated in late‐symptomatic *C9‐500* mice, while *WT* levels remained low. Of note, TLN‐treated *C9‐500* mice displayed lower levels of all three circulating cytokines compared to their age matched untreated *C9‐500* mice (Figure S6C). These data establish that TLN mitigates central and systemic inflammatory signatures in late‐symptomatic *C9orf72*‐linked ALS mice and restores glial homeostasis in the lumbar spinal cord. Together, these multi‐level analyses demonstrate that TLN effectively counteracts key pathological features of ALS, namely oxidative stress, mitochondrial dysfunction, synaptic degeneration, and gliosis, thereby preserving motor neuron health and function.

### 2.8 TLN Treatment Exerts Broad Neuroprotective Effects by Attenuating Key ALS Pathophysiological Features in Familial ALS Mouse Models

**FIGURE 8:**
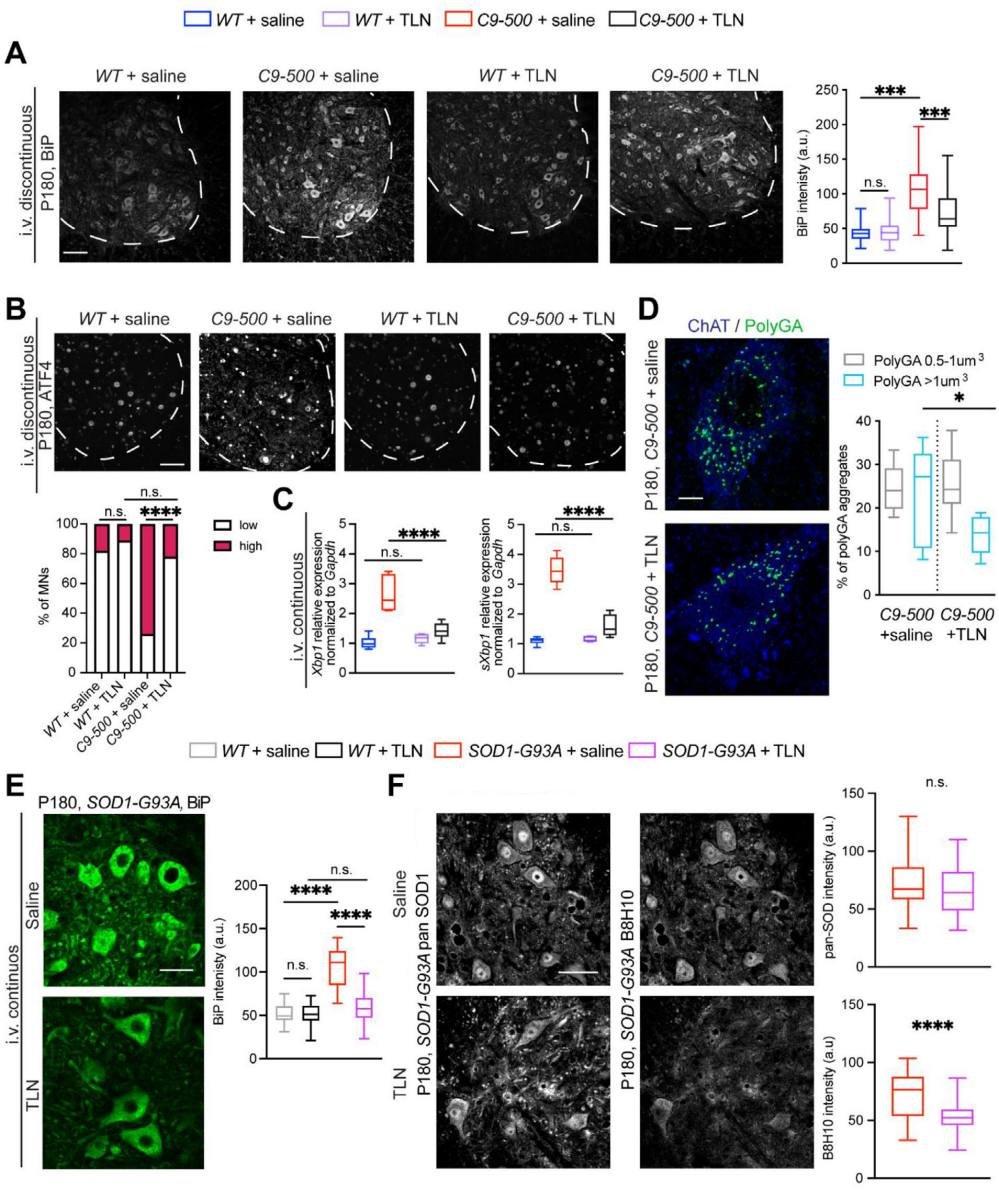
TLN improves clearance of toxic aggregates and restores cellular homeostasis in ALS mouse models. (A) Representative confocal images of the ER stress marker BiP. Q.A. of BiP expression reveal significant decrease in *C9‐500* + TLN i.v. treated animals (One‐way ANOVA: F=159.6, *p* <0.0001; Šidák's multiple comparisons: *WT* + saline vs *C9‐500* + saline ^****^, *C9‐500* + saline vs *C9‐500* + TLN ^****^). N=3 animals/genotype/treatment. Scale bar 100 µm. (B) Representative confocal images of ATF4 expression in the spinal cord. Q.A. of nuclear ATF4 expression reveals significant reduction in nuclear ATF‐4 intensity in *C9‐500* mice after TLN treatment. Chi‐square *WT* + saline vs *WT* + TLN n.s. *p*=0.2278; *C9‐500* + saline vs *C9‐500* + TLN ^****^ *p*<0.0001; *WT* + TLN vs *C9‐500* + TLN n.s. *p*= 0.056. N=3 animals/genotype/treatment. Scale bar 100 µm. (C) Transcript levels measured via q‐PCR for total and spliced *Xbp‐1* (sXbp‐1) which were elevated in *C9‐500* + saline condition and reduced to *WT* levels after TLN treatment. Unpaired t‐test (total *Xbp‐1*): t=4.616, *C9‐500* + saline vs *C9‐500* + TLN, *p*=.001^***^; unpaired t‐test (s*Xbp‐1*): t=9.161, *C9‐500* + saline vs *C9‐500* + TLN, *p*<0.0001^****^. N=6 animals/genotype/treatment. (D) Representative confocal images and 3D rendering of PolyGA in saline and TLN‐treated motor neurons from *C9‐500* animals. Q.A. of PolyGA volume revealed a decrease in aggregates with volumes larger than 1 µm^3^ (One‐way ANOVA: F=7.718, *p*=0.0003; Šidák's multiple comparisons: *C9‐500* + saline vs *C9‐500* + TLN ^*^). N=3 animals/genotype/treatment. Scale bar 20 µm. (E) Representative images of BiP expression in *SOD1‐G93A* animals treated with saline and TLN via i.v. administration. Q.A. displays a significant decrease in ER stress after TLN treatment (One‐way ANOVA: F=103.1, *p* value<0.0001; Šidák's multiple comparisons: *WT* + saline vs *SOD1‐G93A* + saline ^***^; *SOD1‐G93A* + saline vs *SOD1‐G93A* + TLN ^***^). N=3 animals/genotype/treatment. Scale bar 50 µm. (F) Representative confocal immunofluorescence imaging of pan‐SOD1 (detecting total SOD1) and misfolded SOD1 using conformation specific antibody B8H10. *SOD1‐G93A* animals display a decrease in mutant SOD1 protein compared to saline treated animals. Q.A of SOD1 and B8H10 average intensity expression (unpaired t‐test pan‐SOD1: *SOD1‐G93A* + saline vs *SOD1‐G93A* + TLN, t=1.814, *p* value=0.072, n.s.; unpaired t‐test pan‐SOD1: t=1.814, *p* value=0.072; unpaired t‐test B8H10: *SOD1‐G93A* + saline vs *SOD1‐G93A* + TLN, t=6.105, *p* value<0.00001). N=3 animals/genotype/treatment. Scale bar 50 µm.

Since toxic inclusions and impaired cellular homeostasis are key features of most neurodegenerative diseases, we next focused on established dysfunctions such as ER stress and aggregate accumulation in both models [8, 10, 36]. In the *C9‐500* model, confocal imaging revealed that ER chaperone BiP, was expressed at higher levels in the mutant spinal cord, indicative of ER stress and TLN treatment markedly reduced BiP levels in *C9‐500* spinal motor neurons (Figure 8A). Consistent with high BiP levels, ATF4 immunoreactivity was significantly elevated in saline‐treated *C9‐500* mice but was reduced to *WT* levels following TLN administration, further supporting a normalization of the integrated stress response (Figure 8B). qPCR analysis also confirmed that unfolded protein response (UPR), measured via increased expression of total (*Xbp1*) and spliced Xbp1 (*sXbp1*) transcripts in *C9‐500* mice were significantly decreased by TLN treatment, thus restoring ER homeostasis (Figure 8C). Corresponding analysis of DPR; poly(GA) revealed a substantial decrease in the volume of poly(GA) inclusions exceeding 1 µm^3^ in TLN‐treated mutant mice compared to saline‐treated *C9‐500* mice, highlighting the efficacy of TLN in mitigating proteotoxic stress, thus corroborating our proteomic data from mouse and human models (Figure 8D). Importantly, these beneficial effects were not restricted to the *C9‐500* model and in *SOD1‐G93A* mice, TLN administration also led to a significant reduction in BiP expression (Figure 8E). Further analysis using a conformation‐specific antibody that detects pathogenic misfolded SOD1, showed that TLN treatment significantly lowered the levels of misfolded SOD1, while total SOD1 levels, as assessed by pan‐SOD1 immunostaining, remained unchanged (Figure 8F). This selective reduction of toxic SOD1 species suggests that TLN modulates protein quality control pathways to enhance clearance or prevent misfolding, inhibit ER stress and avert toxic protein accumulations. Collectively, these findings underscore TLN's capacity to mitigate multiple convergent ALS‐related pathological processes including but not limited to ER stress, proteotoxicity, and misfolded protein accumulation in both *C9ORF72* and *SOD1‐G93A* models.

## 3 Discussion

In this study, we developed a novel formulation of GM1, TLN, and demonstrated improved pharmacokinetics and ability to cross the BBB. To analyze its potential protective effects in ALS, we first conducted a comprehensive proteomic analyses of disease‐causing mutations in ALS. Using both human and murine models of ALS mutations to ensure robustness, we observed that TLN exerts its therapeutic effects through a multifaceted mechanism that targets key cellular dysfunctions underlying motor neuron degeneration. At the cellular level, TLN modulates mitochondrial dynamics by restoring Ca^2+^ homeostasis and improving energy metabolism. Concurrently, TLN alleviates chronic ER stress by attenuating maladaptive UPR and preventing the accumulation of toxic proteins in two familial ALS models. Collectively, these findings position TLN as a mechanistically driven therapeutic candidate for ALS, supporting its continued advancement toward clinical translation.

### 3.1 Dissecting ALS‐Associated Pathogenic Proteome in Patient iMNs

Identifying causal alterations in ALS is challenging due to the late onset of clinical symptoms, overlapping pathological features, genetic heterogeneity, complex feedback loops, and limited access to early‐stage human neurons. Our study thus leveraged proteomic analyses on human iMNs to identify that ER, mitochondria and synaptic compartments are significantly altered in mutant human iMNs. TLN treatment not only counteracted these prominent impairments but also modulated the expression of proteins associated with the ER‐mitochondria and ER‐Golgi membranes. For example, in *C9ORF72*‐ALS, toxic DPRs, particularly Poly‐GA and Poly‐GR, accumulate at mitochondria‐associated membranes (MAMs) and mitochondria, disrupting ER‐mitochondria tethering, impairing Ca^2+^ signaling, and reducing ATP production [10, 37, 38]. These DPRs also activate PERK and IRE1 pathways of the UPR, promoting sustained ER stress and neurotoxicity [39, 40]. Similarly, mutant SOD1 localizes to mitochondria and MAMs, impairing respiratory chain function, increasing ROS, and disrupting VAPB–PTPIP51‐mediated ER‐mitochondria contacts, leading to Ca^2+^ dysregulation and bioenergetic failure [41, 42, 43]. Mutant TDP‐43 alters mitochondrial dynamics and oxidative phosphorylation, while also inducing ER stress and UPR activation [44, 45, 46, 47]. Lastly, FUS mutations similarly impair mitochondrial transport, disturb ER‐mitochondria tethering, and disrupt Ca^2+^ homeostasis [48, 49]. Our study provides compelling evidence that therapeutic compounds targeting ER, mitochondria and synaptic processes are fundamental for neuroprotection as well as for symptomatic stage amelioration of ALS symptoms.

### 3.2 Mode of Action of TLN and Neuroprotection via Mitochondrial and Synaptic Pathways

GM1 has been shown to amplify neurotrophic response, block excitotoxicity and promote neurite growth in rat models of Parkinson's disease and in cultured *SOD1‐G93A* neurons, suggesting a protective role in neurodegeneration [18, 26, 50]. GM1 is found predominantly in the outer layer of neuronal cell membranes, mainly at lipid rafts and is concentrated in synaptic plasma membrane microdomains, where GM1 plays critical roles in neuroprotection, neuroplasticity, and repair [51]. Crucially, its localization on the cell membrane enables GM1 to enhance the action of neurotrophic factors, especially nerve growth factor (NGF) either by promoting TrkA receptor phosphorylation or facilitating the clustering of receptors like TrkA and EGFR and activation of downstream survival signaling pathways [52]. Notably, TLN‐Dil accumulates within neurons, indicating that TLN likely exerts additional neuroprotective effects intracellularly, thus distinguishing its neuroprotective mechanism from classical GM1. Supporting this unique mode of action, TLN specifically restores mitochondrial Ca^2+^ uptake, counteracting functional mitochondrial Ca^2+^ uptake deficits in human ALS patients iMNs, an effect not observed with GM1 or BVs alone. Importantly, our study also revealed that TLN ameliorated overall cellular proteostasis and improved toxic aggregate handling in rodent models of familial ALS (Figure 8D,F). However, it is also plausible that internalized TLN may directly interact with these aggregation prone proteins and inhibit their aggregation, similar to GM1's documented ability to bind synaptic membrane localized α‐synuclein and prevent its self‐aggregation and fibril formation [53]. Future NMR‐based studies are required to assess the direct binding of TLN to PolyGA or misfolded SOD1 proteins / aggregates, nevertheless, proteomic analyses of rodent spinal cord also implicate indirect TLN‐linked mechanisms such as improved ER homeostasis and increased ubiquitin enzymes, which collectively could counteract protein accumulation and aggregation.

TLN treatment positively impacted the expression of SNARE interactors and complexes within human iMNs and rodent motor neurons (Figure 2D), highlighting its mode of action on the synapse. This aligns with previous observations of SNARE dysfunction at the NMJ driving early motor‐unit failure in ALS and related TDP‐43 proteinopathies. Early presynaptic defects involving reduced expression of SNAP‐25 and syntaxin‐1, and imbalances in Munc18‐1/SNAP‐25 phosphorylation at the NMJ of *SOD1‐G93A* mice, have been implicated in disease onset and progression [54, 55]. In the case of TDP‐43, loss of function causes cryptic exon inclusion in UNC13A, triggering nonsense‐mediated decay and loss of UNC13A protein, disrupting Munc13‐dependent SNARE priming and weakening transmitter release at the presynapse [56]. Consistently, transcriptomic analysis of post‐mortem cortical tissue from patients with TDP‐43 pathology as well as sporadic ALS patients show significant alterations in SNARE‐related proteins and molecules involved in synaptic vesicle trafficking, and calcium‐dependent exocytosis/endocytosis [57, 58]. In this regard, the therapeutic action of TLN on the SNARE pathway holds promise in ameliorating key disease drivers across both familial and sporadic ALS.

Crucially, TLN treatment normalized the expression of TAP complex and major histocompatibility complex (MHC) class I (Figure 3C,D) and blunted pro‐inflammatory responses (Figure S6D). Notably, TAP proteins are members of the ABC transporter family, playing a critical role in the processing and presentation of MHC class 1 restricted antigens. Numerous studies have implicated neuronal MHC‐1 in synaptic plasticity, brain development, and axonal regeneration [59, 60, 61]. More recent findings suggest that neuronal MHC‐1 expression contributes to neuroinflammatory processes and immune‐mediated neurodegeneration. While TAP complex involvement has not been directly studied in ALS, MHC‐1 is expressed in spinal motor neurons, particularly in their axons and at NMJs, and upregulated in response to injury. Thus, it is plausible that TLN treatment exerts beneficial effects in part by reducing expression of TAP and MHC class 1‐driven inflammatory responses in ALS. Additionally, abnormally high levels of IL‐6 and IL‐1β directly correlate with disease severity and progression in familial and sporadic ALS patients [35], both of which were significantly reduced in TLN treated mice, highlighting the overall neuroprotective potential of TLN.

### 3.3 Altered GM1 Levels in ALS and Parkinson Patients

Abnormal ganglioside composition in ALS has been reported as early as 1980s, along with reports of additional complex and unusual gangliosides found in ALS patient brains, spinal cords and CSF [62, 63, 64]. In 2015, Dodge et al. reported increased levels of GM3 and GM1, along with increased activity of hexosaminidase in the spinal cords of ALS patients and end‐stage *SOD1‐G93A* mice [23]. Interestingly, longitudinal analysis in *SOD1‐G93A* mice revealed significantly reduced GM1 levels at presymptomatic and symptomatic stages suggesting that reductions in GM1 levels might selectively compromise motor neuron function and viability in ALS [23]. Further, intracerebroventricular delivery of GM3 significantly delayed the onset of paralysis and extended survival of *SOD1G93A* mice [23]. The promising results from our study, showing neuroprotective effects in preclinical models of ALS, suggests that GM1 or GM3 interventions might lead to favorable outcome in ALS patients. Similarly, GM1, GD1a and GT1b are reduced in the substantia nigra of male PD patients with elevated glucosylceramide in PD brain tissue [65, 66], supporting a causal and conserved role for ganglioside imbalance in neurodegenerative disease onset and progression [66, 67].

### 3.4 BBB and Challenges in ALS Drug Development

The BBB presents a major hurdle in the development of effective therapeutics for ALS. In the context of ALS, this challenge is further amplified by regional heterogeneity in BBB integrity and its potential disruption during disease progression, which may alter drug distribution and efficacy. Several drug delivery systems that aim to enhance therapeutic efficacy by improving bioavailability, crossing the BBB, and ensuring targeted delivery to affected neuronal populations are currently being explored, with nanoparticles being one such approach. Liposomes are ideal drug delivery systems, due to ease of preparation, low toxicity, and high bioavailability. Their ability to encapsulate both hydrophilic and lipophilic compounds makes them especially suitable for brain‐targeted delivery. However, their clinical utility is limited by rapid clearance via the reticuloendothelial system which reduces their circulation time and delivery efficiency [68]. Thus, to achieve prolonged liposome circulation, size reduction to the nanometer scale and surface modification with polyethylene glycol have been employed to avoid clearance by the immune system [69]. Antioxidant, astaxanthin‐loaded nanostructured lipid carriers (AST‐NLCs) were successfully delivered to the brain in a rat model of Alzheimer's disease [70]. In the recent ongoing NEON Phase I open‐label trial, 12 Parkinson's disease patients received weekly i.v. infusions of TLN for two months, with a maximum tolerated dose of 720 mg. Notably, patients showed significant improvements in motor function and quality of life, as reflected by reductions in MDS‐UPDRS and PDQ‐39 scores, without changes in their regular dopaminergic medication [31]. While long term TLN toxicity studies in humans are in progress, our data from chronically TLN‐treated mice reveals that it is well tolerated with no adverse effects such as weight loss or reduced liver or kidney function. In the future, a safety assessment in ALS patients may directly be conducted in a phase 1a/b trial. Overall, the favorable outcomes of TLN in preclinical ALS rodent models and human‐patient derived neurons, suggest that TLN is a promising therapy for ALS patients.

## 4 Conclusions

Our proteomic data highlights significant deficits in multiple cellular and biochemical processes within *C9ORF72*‐ALS patient‐derived MNs and conserved impairments are present within rodent models of ALS. Further, our study provides evidence that TLN acts through a multifactorial mechanism, modulating key intracellular dysfunctions consistently observed across familial ALS models, including *C9ORF72* and *SOD1* mutations. At the mitochondrial level, TLN restores Ca^2+^ homeostasis and improves bioenergetic function, which are often impaired due to disrupted ER‐mitochondria communication and defective mitochondrial Ca^2+^ uniporter complex (MICU) activity, a hallmark of ALS pathology [8, 43]. Concurrently, TLN alleviates chronic ER stress, a known contributor to ALS progression, by downregulating ER stress markers such as BiP. These effects suggest that TLN actively contributes to the re‐establishment of ER proteostasis, a pathway disrupted in both familial and sporadic ALS [71]. However, the mechanisms behind the broad pleiotropic effect of TLN remain unclear. A crucial clue might lie in the assessment of TLN distribution within various cellular compartments. Furthermore, the evaluation of GM1 interaction with mutant ALS causing proteins will shed light into the mechanism related to resolving /reducing cellular aggregate buildup and dampening neuroinflammation. Given TLN's involvement in modulating mitochondria Ca^2+^ uptake, and function, influencing ER‐mitochondrial properties, synaptic function and protein folding regulation, TLN harbors the potential to mediate diverse, context‐dependent effects across neurodegenerative and neurological disorders. Furthermore, TLN may act through additional protective mechanisms, especially linked to its intracellular localization, and those effects distinguish further the neuroprotective properties of TLN from free GM1. In 2021, TLN was granted the orphan drug designation for ALS by the European Medical Agency (EU/3/21/2426) and FDA, setting the stage for future human clinical trials, enabling further mechanistic insight into the disease process and highlighting precise neuroprotective pathways. The consistent reduction in GM1 levels in both patient samples and animal models underscores TLN as a promising therapeutic candidate for ALS, though the exact molecular pathways remain to be fully elucidated.

## 5 Materials and Methods

### 5.1 Formulation of Talineuren (TLN)

TLN is a liposomal formulation of the active pharmaceutical ingredient (API) GM1. The base vesicle, hence, the carrier liposome consists of cholesterol and sphingomyelin. It is provided as a concentrated liposomal suspension in phosphate buffered solution. The potency of TLN is based on the API GM1 content 6 mg/ml. The TLN dosages used in the different experiments are always expressed in terms of GM1 content and concentration (e.g. 7.5 mg/kg of TLN means 7.5 mg/kg of the API GM1 in the liposome). Three different batches were used for the presented study. The identical material was used in human clinical investigations (NEON Phase I trial, NCT04976127). For TLN dosage, frequency and administration route refer to Table 2.

**TABLE 2:** Depicting Talineuren (TLN) dosing regimes, frequency and route of administration.

TLN Formulation: 6mg of GM1/mL										
**Species**	**Admin. route**	**Fequency**	**Age**	**TLN: API concentration (GM1)**	**Free GM1**	**Base vesicles**	**DiR (10 µg/mL)**	**DiR (10 µg/mL)**	**Analsyses**	**Figures**
Rat	i.v.	daily for 4 days	N/A	12.3 mg/kg	12.3 mg/kg				Biodistribution analysis	Figure 1A,D

**TLN Formulation: 6mg of GM1/mL**										
**Species**	**Admin. route**	**Fequency**	**Age**	**TLN: API concentration (GM1)**	**Free GM1**	**Base vesicles**	**DiR (10 µg/mL)**	**DiL (10 µg/mL)**	**Analsyses**	**Figures**
Mouse	i.v.	single injection	N/A	15mg/kg‐DiR			10mL/kg		Bodistribution analysis	Figure 5A–C
Mouse	i.v.	single injection	N/A	15mg/kg‐ Dil				10mL/kg	Bodistribution analysis	Figure 5D
Mouse	i.v.	alternate day	P100‐P180	15 mg/kg					Pathological hallmarks	Figures 6, 7, 8A–F, Figures S5 and S6A
Mouse	Drinking water	daily	P100‐P180	7.5 mg/kg					Early Pathological hallmark	Figure 6, 7, Figures S2,S3,S6B–D
Mouse	Drinking water	daily	P180‐endstage	15 mg/kg					Pathological hallmarks	Figure 6, 7, Figures S2,S3, and S6B–D
Mouse	Drinking water	Acute 72h	N/A	30 mg/kg					Biodistribution analysis	Figure 1E

**TLN Formulation: 6mg of GM1/mL**										
**Species**	**Admin. route**	**Fequency**	**Age**	**TLN: API concentration (GM1)**	**Free GM1**	**Base vesicles**	**DiR (10 µg/mL)**	**DiR (10 µg/mL)**	**Analsyses**	**Figures**
Human iMNs	in vitro	Single	N/A	15 µg/mL					Internalization (24h)	Figure S1A
Human iMNs	in vitro	Single	N/A	5, 10 & 15 µg/mL					Dosage/timeline calculation	Figure S1B
Human iMNs	in vitro	Single	N/A	15 µg/mL					Mass Spec (48h)	Figures 2 and 3
Human iMNs	in vitro	Single	N/A	15 µg/mL	15 µg/mL				Glutamate tox. (24h & 48h)	Figure 4B
Human iMNs	in vitro	Single	N/A	15 µg/mL	15 µg/mL	2.5 ml			Mito.Ca2+imaging (24h)	Figure 4D; Figure S1C
Human iMNs	in vitro	Single	N/A	15 µg/mL					Sea horse assay (24h)	Figure 4E

### 5.2 Characterization of the Formulation

GM1 ganglioside, eSM and cholesterol content in TLN were measured in‐house by RP‐UHPLC‐CAD. Concurrent quantification of lipids is based on the HPLC‐ELSD method proposed by Shibata (Shibata et al., 2013). The method was adapted by replacing ELSD by CAD to increase the dynamic range. Initial disruption of liposomes i.e. dissolution of lipids is achieved by dilution of the drug product sample in methanol. Lipids are subsequently separated using a C18 column with a gradient mobile phase consisting of ammonium acetate buffer and ammonium acetate in methanol. The method for determination of free micellar (extraliposomal) GM1 ganglioside is measured by size exclusion (SEC)‐UHPLC‐DAD. Free micellar GM1 is separated from liposomal GM1 by a size exclusion column (100 nm pore size and 5 µm particle size) through which an isocratic flow of aqueous mobile phase consisting of PBS (pH 6.8) is applied at a rate of 0.2 mL/min. UV‐absorption at 210 nm is used to detect both the micellar and liposomal GM1. Average liposome size, size distribution and polydispersity index (PDI) are measured by dynamic light scattering. Size and PDI were measured in samples diluted 1:10 in distilled water, for zeta potential the sample was diluted 1:50. Characterization of liposomes using cryo‐TEM was performed by Vironova. Samples were diluted 1:10 in PBS buffer and mounted on continuous carbon grid. Images were acquired with GLACIOS‐9953104 TEM and analyzed by Vironova's software.

### 5.3 iPSC Differentiation into Motor Neurons (iMNs)

The healthy and *C9ORF72*‐ALS iPSCs were obtained from iPSC bank (Biomedicum Stem Cell Center, GoEditStem platform, HiLIFE, Helsinki, Finland and the iPSC Core, Cedar Sinai, USA). iPSCs were cultured in GeltrexTM (ThermoFischer) coated plates in mTeSR^TM^1 (StemCell technologies) media. Motor neuron differentiation was performed as previously described [72, 73] with some modifications. Human iPSCs were dissociated to single cells using Accutase (StemCell technologies) and seeded at 3X10^6^ onto 10 cm plate with N2B27 differentiation medium (Advanced DMEM/F12:Neurobasal (1:1) medium, 1% Pen/strep (Gibco), 1% GlutaMAX (Gibco), 0.1 mM 2‐mercaptoethanol (Gibco), 1X B27 supplement (Gibco), 1X N2 supplement (Gibco), supplemented with 10 ng/mL basic fibroblast growth factor (StemCell technologies), 20 µM SB431542 (StemCell technologies), 0.1 µM LDN193189 (StemCell technologies), 3 µM CHIR99021 (StemCell technologies), 10 µM L‐Ascorbic Acid (L‐AA; Sigma) and 1X Revitacell supplement (Gibco)) to initiate the formation of embryoid bodies (EBs). On day 2, patterning of EBs was induced by the addition of media supplemented with 100 nM all‐trans retinoic acid (RA; sigma) and 500 nM Smoothened Agonist (SAG; StemCell technologies). EBs were pelleted and fed with fresh media on every alternate day until day 14. 10 ng/ml Brain derived neurotrophic factor (BDNF; StemCell technologies) was added from day 7 while 10 µM DAPT (StemCell technologies) was added from day 9. EBs were dissociated using trypsin on day 16 and triturated with ice cold cell trituration and wash medium (1X PBS (Gibco), 0.45% Glucose, 0.1% Bovine Serum Albumin (BSA; Sigma), 2 mM MgCl2, 0.8 mM EDTA (Invitrogen), 2.5% Fetal Bovine Serum (FBS; Sigma), 1X N2 supplement, 1X B27 supplement and DNAse). Triturated EBs were then plated on poly‐ornithine/laminin (Sigma) coated plates in motor neuron feeding medium (Neurobasal medium (Gibco), 1X glutaMAX, 1X Non‐essential amino acid (NEAA, Gibco), 0.1 mM 2‐mercapthoethanol, 1X N2 supplement, 1X Pen/strep, 1X B27 supplement, 10 ng/mL glial cell derived neurotrophic factor (GDNF; StemCell technologies), BDNF 10 ng/ml, 10 ng/ml insulin‐like growth factor (IGF‐1; StemCell technologies), 10 ng/mL Ciliary neurotrophic factor (CNTF; StemCell technologies), 100 nM RA and 10 µM AA and kept at incubator at 37°C and 5% CO2 for further maturation.

### 5.4 Rodent Strains

The *C9‐500* BAC mouse line (*FVB/NJ‐Tg(C9orf72)500Lpwr/J*) carrying a human *C9ORF72* gene under a human promoter with ∼500 hexanucleotide repeats described in [74, 75] was purchased from Jackson Laboratory (RRID: IMSR_JAX:029099) and kept in heterozygosis crossed with (non‐carrier) mice *FVB/NJ* (Janvier labs, *SC‐FVBN‐F*). Our colony displays an acute phenotype, which is observed in 25%–30% of females with a median life span of 105 days whereas the remaining female and male mice exhibit a slow‐progressing phenotype with females having a median life span of approximately 250 days and males 260 days. Long‐range PCR was regularly done to identify repeat length‐matched cohorts. The moderate expresser transgenic mice line of human *SOD1* carrying a Gly93‐Ala mutation (*B6SJL‐Tg(SOD1‐G93A)dl1Gur/J*), crossed into *C57BL/6J* background were purchased from Jackson Laboratory (RRID:IMSR_JAX:002726). SKH1 mice and Sprague Dawley (CD‐1) rats were purchased from Charles River Laboratories.

Animal care, housing, ethical experimental usage, and procedures were in accordance with the Swiss Veterinary Law and University of Missouri guidelines. The study was approved by the Animal Commission of Canton of Bern, Switzerland and IACUC # 53562, University of Missouri, Columbia, USA.

### 5.5 Hindlimb Clasping Measurements

Hindlimb clasping analyses was performed as described in [75]. Among the slowly progressing *C9‐500* animals, male and female mice showed gradually worsening clasping phenotypes. For clasping measurement and scoring the tails of the mice were grasped from the base and the mice were lifted for 20 seconds (s). The position needs to be maintained for 20 s and failure to do so results in the termination of the test. If both hindlimbs are consistently splayed outward, away from the abdomen it is assigned a score of 0. If only one of the hindlimbs is retracted near the abdomen for most of the time, the score assigned is 1. If both hindlimbs are retracted close to the abdomen, the animal receives a score of 2. Finally, close to end stage animals that show both hindlimbs tightly clinched to the abdomen when they are lifted are assigned a score of 3.

### 5.6 Hydration Ratio Measurement using EchoMRI

Mice were removed from their home cages and placed individually into secured body‐composition holding tubes to minimize movement during imaging. Before each session, the EchoMRI Analyzer was calibrated using a standard 34‐g canola oil calibration tube, and a system test was performed via the EchoMRI software to verify instrument performance. Once the mouse was settled in the tube, a whole‐body composition scan was initiated, with scan durations ranging from approximately 30 s to 3 min depending on the selected precision settings. After scanning, mice were removed from the tube and returned to their cages, and the resulting data were saved and processed to quantify lean mass, fat mass, and total body water. Hydration ratio in % was calculated as (Total water—free water) / lean mass [76].

### 5.7 Pharmacological Treatments of iMNs and Mice

Mature iMNs were treated with TLN, free‐GM1 and BV, at a concentration of 15 µg/mL for 24 h or 48 h. Animals from both groups control and TLN were weighed and accordingly received a single intravenous (i.v.) injection of saline (0.9% NaCl) in the lateral tail vein or TLN at a dosage of the API GM1 of 15 mg/kg/2 days. For oral treatment TLN was diluted in drinking water to reach a dosage of the API GM1 of 7.5 mg/kg/day until P180, after this age, when mice converted to a stronger symptomatic phenotype, the dosage of TLN was doubled to 15 mg/kg/day in 1% sucrose and stevia to mask the saltiness of the compound. Drinking water was refreshed regularly, with fresh TLN added every 2–3 days to maintain consistent dosing.

### 5.8 TLN Dosage Calculation for iMNs

iMNs at day in vitro (DIV) 10 were treated TLN at the dose of 5, 10 and 15 µg/mL for 24, 48, and 72 h. Live/Dead assay was performed according to the kit protocol (ThermoFischer, L3224) to visualize and plot the percentage of alive cells in each experimental condition.

### 5.9 Glutamate Toxicity Assay on iMNs

iMNs at DIV 5 were treated with free GM1 or TLN at the dose of 15 µg/mL for 24 h or 48 h. In the last 8 h of treatment glutamate was added at a concentration of 10 µM. iMNs were incubated with Propidium Iodide (Stemcell, 75002) for 10 min to visualize dead cells.

### 5.10 Immunofluorescence on iMNs

iMNs plated on coverslips were fixed using 4% paraformaldehyde PFA for 15 min and blocked for 1 h with 3% bovine serum albumin (BSA) and 0.1% TritonX‐100 in phosphate buffered saline PBS. After blocking, neurons were incubated with the following primary antibody goat anti‐ChAT (Millipore, AB144P, 1:500), chicken anti‐MAP2 (Sigma Aldrich, AB15452, 1:500) in blocking buffer overnight at 4°C. After washing three times with PBS, cells were incubated in blocking buffer with Alexa Fluor fluorescently labeled secondary antibodies and DAPI for 1 h at room temperature. Cells were then washed with PBS and mounted on glass slides using DAKO (Dako north America, S3023) mounting media.

### 5.11 Measurement of mitochondrial activity from iMNs

Mitochondrial respiration was monitored via oxygen consumption rate (OCR), using Seahorse XFp instrument coupled to a Seahorse XF Cell Mito Stress Test Kit (Agilent). iPSCs were plated and differentiated to iMNs on Seahorse 96 well plates pre‐coated with Matrigel. Culture media was changed with Seahorse DMEM basal media 45 min before the measurements. Seahorse ports were filled with 1 µM oligomycin, 1 µM FCCP, 0.5 µM of rotenone and antimycin A. OCR in the different respiration states was accessed with Wave 2.3.0 software (Agilent). Seahorse Flux Pak cartridges were filled with the reagents at 10‐fold concentration: (A) ADP: 40 mM; (B) oligomycin: 25ug/mL; (C) FCCP: 40 µM; (D) antimycin: 40 µM.

### 5.12 Mitochondrial Ca^2+^ Uptake Measurements

Mitochondrial Ca^2+^ uptake measurements were adapted from McKenzie et al. [77]. Briefly, neurons were incubated for 45 min in staining solution: 156 mM NaCl, 3 mM KCl, 2 mM MgSO4, 1.25 mM KH2PO4, 10 mM D‐glucose, 2 mM CaCl2 and 10 mM HEPES pH 7.35, 5 µg/mL (w/v) Fluo‐4, AM, 10 µM Verapamil. After a brief wash with Ca^2+^ free HBSS, cells were incubated for 10 min with intracellular solution: 6 mM NaCl, 130 mM KCl, 7.8 mM MgCl2, 1 mM KH2PO4, 0.4 mM CaCl2, 2 mM EGTA, 10 mM HEDTA, 2 mM malate, 2 mM glutamate, 2 mM ADP, 20 mM HEPES pH 7.1, 25 µg/mL (w/v) digitonin and 1 µM thapsigargin. Images were acquired every 20 s using 40X water immersion objective fitted to Fluoview 1000 (Olympus), the first 60 s considered as baseline, followed by depolarization of neuron with 50 mM KCl. Images were analyzed using Fiji, multiple regions of interest (ROI) were chosen inside the cytosol of each motor neuron and fluorescence intensity was calculated over different time frames. To calculate ΔF, the median intensity values were divided by the average of the first 60 s of recording (F0) per single ROI.

### 5.13 RNA Extraction and qPCR

Total RNA was isolated from mouse spinal cord tissue using a phenol–chloroform based extraction method according to established protocols [78]. Extracted RNA was quantified and assessed for integrity prior to downstream analyses. cDNA was made using GoScriptTM Reverse transcriptase (A5000, Promega). qPCR was performed with HOT FIREPol EvaGreen qPCR Mix Plus (Rox) (Solis Biodyne, 08‐24‐00008), using an Applied Biosystem 7500 Real‐Time PCR systems. RNA levels were normalized to *Gapdh* and gene expression differences were quantified with the comparative CT method. Primer sequences are shown below.

**Table:** 

		Mouse primer sequences		
*Gapdh*	Forward	CATCACTGCCACCCAGAAGACTG		
	Reverse	ATGCCAGTGAGCTTCCCGTTCAG		
				
*Xbp1*	Forward	TGTCCATTCCCAAGCGTGTTCT		
	Reverse	TGGAGCAGCAAGTGGATTT		
				
*sXbp1*	Forward	AAGAACACGCTTGGGAATGG		
	Reverse	CTGCACCTGCTGCGGAC		

### 5.14 Immunohistochemistry

Mice were transcardially perfused with 4% paraformaldehyde (PFA) in 1X phosphate buffered saline (PBS); brain and lumbar spinal cord were isolated and kept overnight at 4°C in the same fixative solution, followed by cryoprotection in 30% sucrose/PBS until use. After embedding, 50 µm spinal cords sections and 30 µm brain sections were cut using a cryostat. Antibodies used for immunofluorescence were: mouse anti‐KDEL/BiP (1:500, Enzo Life Science, SPA‐827), mouse anti‐8‐OHdG (1:1000, ABCAM,15A3), rabbit anti‐GRP78/BiP (1:500, Abcam, ab21685); goat anti‐ChAT (1:500, Millipore, AB144P), rabbit anti‐SOD (1:200, Enzo LifeScience, ADI‐SOD‐100‐F), mouse‐anti B8H10 (1:500 Medimabs), rabbit anti‐ATP5j (1:500, Invitrogen, PA5‐29202), rabbit anti‐ATF4(1:500, Abcam, ab7269), guinea‐pig anti‐IBA1 (1:500, SYSY, 234 308), rabbit anti‐GFAP (1:500, Abcam, ab216839). Heat‐mediated antigen retrieval was performed using Sodium citrate buffer 10 mM pH 6 for ATP5j staining. Sections were incubated in the buffer for 20 min at 85°C in a thermocycler, followed by 2 PBS washes for 10 min each. Sections were kept for 2 h in PBS solution containing 0.05% Triton X‐100 and 10% normal donkey serum (NDS, Jackson Immunoresearch) after the antibodies were applied in PBS, 3% NDS, 0.05% Triton X‐100, and incubated for two nights at 4°C. Sections were then briefly washed with PBS and incubated for 2 h at room temperature (RT) with appropriate combinations of secondary antibodies from Invitrogen. Sections were mounted on a glass slide, and coverslips were mounted with Dako (Dako north America, S3023) fluorescent mounting media. Spinal cord sections for immunohistochemistry were processed as follows: sections were treated with heat‐mediated antigen retrieval using sodium citrate buffer 10 mM pH 6.0 and immersed in 3% H2O2 in PBS for 20 min to block endogenous peroxidase activity. This was followed by a blocking step in PBS containing 0.05% Triton X‐100 and 10% normal donkey serum (NDS) and incubated overnight at 4° with goat anti‐Neun (1000, Millipore, MAB377) diluted in the same blocking solution. The next day the sections were incubated with the appropriate biotinylated secondary antibody (1:500) followed by 1 h incubation in PBS solution containing biotin‐avidin complex (1:100, Vector Labs), finally, the 3,3′‐diaminobenzidine (DAB) reaction was developed. The glass slides were dehydrated via ascending concentrations of ethanol and rinsed in xylene before being cover slipped. Images were acquired using an Olympus microscope (BX51).

### 5.15 Image Analysis

To assess the biodistribution of TLN‐Dil sections were directly mounted on a glass slide and imaged at the confocal microscope.

For the analysis of BiP, B8H10 and pan‐SOD1 labeling intensity, data were acquired using identical confocal settings, with signals at the brightest cells being non‐saturated, and background levels outside motor neuron pools still detectable. Images were analyzed quantitatively using FiJi, intensity values below 50 arbitrary units (a.u.). are considered as baseline expression levels. Signal intensity values for the antigen of interest in alpha motor neurons were calculated per animal over five consecutive images of the ventral horns of the lumbar spinal cord and primary motor cortex (Layer V) Z‐stack spaced 0.5 µm, after background subtraction. Imaris software was used to reconstruct the 3D isosurface for PolyGA to assess the volume of the aggregates. The analysis of 8‐OHdG staining was done using Fiji where three consecutive Z stacks were taken into consideration for the maximum projection, motor neurons were manually outlined as ROI and average intensity was calculated. For analysis of ATP5j staining Imaris software was used to reconstruct the 3D isosurface of the mitochondria per motor neuron considering 4 consecutive Z stacks. The intensity values were then arbitrary divided into low medium and high category. Intensity values below 70 (a.u.) were considered low, values between 70 and 140 were considered medium and values exceeding 140 were considered high intensity.

For the analysis of Iba1 staining, 15 Z‐stacks spaced 0.5 µm apart were merged into a maximum‐intensity projection. The Neuroanatomy plugin in Fiji, which includes integrated Sholl analysis, was used to quantify microglial ramification. The center of the cell body was used as the reference point for the measurements, and radii were spaced 1 µm apart from this center. The number of intersections per radius was then plotted using GraphPad Prism. For the analysis of GFAP staining, five Z‐stacks spaced 0.5 µm apart were merged into a maximum‐intensity projection. An ROI with an area of 38 099.353 µm^2^ was selected in the ventral horn, and the thresholding function was used to segment the GFAP‐positive signal. The occupied GFAP‐positive area within the ROI was then measured.

### 5.16 Qualitative Biodistribution by In Vivo and Ex‐Vivo Analyses

SKH1 mice were randomly distributed and received a single product administration. Product samples were prepared for a final concentration of 10 µg/mL of DiR, irrespective of free DiR or TLN‐DiR. For TLN‐DiR: 6 mg/mL TLN was supplemented with 10 µg/mL DiR by using ultrasonic waves for 10 min at medium strength for proper membrane insertion of the DiR. Of these samples: 10 mL/Kg were administered i.v. to conscious animals with a disposable plastic syringe of 1 mL and a 26G needle. The volume to be injected was rounded to the nearest 10 µL. In vivo fluorescence acquisitions were performed at: D00 (directly after injection), D00+4 h (4 h), D01 (24 h) and D02 (48 h). Acquisitions on D02 were followed by organs sampling and an ex‐vivo acquisition. For each fluorescence time step, a fluorescence acquisition was performed on a naive uninjected SKH1 mouse to assess background fluorescence level. On D00, fluorescence acquisitions were performed with the optical imaging system IVIS Spectrum of Perkin Elmer. 2D fluorescence imaging was performed by sensitive detection of light emitted by fluorescent dyes (DiR dye in this study). In vivo fluorescence acquisitions were performed on anesthetized mice. Mice were induced and maintained under anesthesia with a mix of Isoflurane (1%–3%) and oxygen (1–2 L/min). During in vivo acquisitions on D00, D01 and D02, the animals were placed in dorsal and ventral recumbency. For all in vivo acquisitions, particular attention was taken into the animal placement to enable the calculation of the fluorescence signal in brain, spinal cord, liver and spleen. Parameters of in vivo fluorescence imaging were: field of view 13×13 cm, fluorescent label DiR, excitation wavelength 745 nm, emission wavelength 800 nm, exposure time was set automatic for a maximum of 20’000 counts.

Animals were euthanized under volatile anaesthesia (Isoflurane 1%–5%; oxygen 1‐2 L/min) by cervical dislocation at D02. Animal's death was checked before corpse elimination. After euthanasia, the brain, spinal cord, liver and spleen of each animal were sampled. The brain was then cut into 5–8 mm thick slice using a scalpel in the longitudinal way. Ex‐vivo acquisitions were performed on each animal's brain, spinal cord, liver and spleen. Ex‐vivo acquisitions were performed using the same imaging system and parameters than for the in vivo fluorescence. The fluorescence acquisitions were analyzed with the Living Image software 4.5.4 version. To calculate the fluorescence signal in brain, spinal cord, liver and spleen, a Region of Interest (ROI) was placed on each organ in the ventral and dorsal images. The ROI size was adapted to each organ and was fixed once for all for each organ of interest. All the fluorescence images were radiance efficiency thresholded in order to preserve, in the in‐vivo and ex‐vivo images, only high radiance intensity pixels relative to the fluorescent product. The fluorescence signal corresponding to the total radiance efficiency (expressed in [p/s]/[µW/cm^2^]) was measured in each ROI. The total radiance efficiency throughout the study was compared between products. Background signal was determined in each ROI on uninjected mice serving as background mice.

### 5.17 Quantification of Brain GM1 After Acute Oral Administration: Sample Processing and Analytical Workflow

Mice were given 30 mg/kg TLN in drinking water diluted with Stevia for 72 h. Brain extracts and standards were stored at ‐20C. 5.5 mg of brain were homogenized in 275uL of 50% methanol using a Potter‐Elvehjem homogenizer tube for 10 min and then spun down with a tabletop centrifuge. 200ul of the liquid fraction was used for measurement. Standard Curve: The standard curve was plotted from GM1 (GM1 (SIGMA, G9652‐1MG), C18 Ganglioside GM1‐d3 (d18:1/18:0‐d3) (ammonium salt); Cayman Chemical. Two transitions (precursor>fragment pairs) were used to quantify GM1. The 772>290 transition was published [79] and a second one, 772>83, was determined using Waters IntelliStart analysis of pure compound. Both transitions showed good linearity, R^2^>0.99.

### 5.18 Ultra‐Performance Liquid Chromatography (UPLC)

Samples and standards (10 uL injections in triplicate) were separated using an Acquity H‐class UPLC (Waters) on a Phenyl‐Hexyl column (3.5um×2.1 mm×50 mm) by gradient delivery (0.25 mL/min) of solvent. Mobile phase A: 0.028% ammonium hydroxide in water; Mobile phase B: 0.028% ammonium hydroxide in methanol. NOTE: the Gobburi method was shortened to a total run time of 5.5 min as only a single compound was being examined.

### 5.19 Quantitation using Target Lynx

A GM1‐specific peak picking and integration method was developed using the retention time data for a mid‐point standard (250 ng/mL). Area under the curve (AUC) for GM1 and d3GM1 was calculated by the software using default parameters. Two parameters (peak shape and shoulder) were employed;integrate peak +/− 0.1 min and ignore shoulder peaks. Data were exported to excel, and the linear response equation was calculated.

### 5.20 Mass Spectrometry

For iMNs cells media was removed from the plate and cells were rinsed with DPBS (w/o Ca^2+^ and Mg^2+^) to remove any media residuals. Cells were collected using a cell scraper and store at −80 until use. For *C9‐500* mice animals were anesthetized using isofluorane and decapitated, spinal cords were collected, and the ventral horn of the lumbar region was dissected under a stereo microscope. The collected tissue was stored at ‐80 until use. Tissue was homogenized using a FastPrep homogenizer before protein extraction.

Cell pellets were lysed in 8 M urea/100 mM Tris, containing proteases inhibitor cocktail (Complete EDTA free, Roche, Rotkreuz), reduced, alkylated and precipitated with acetone at ‐20°C overnight. The pellet was re‐suspended in 8 M urea/50 mM Tris pH8 and protein concentration was determined with Qubit Protein Assay (Invitrogen by Life technology, Zug, Switzerland). An aliquot corresponding to 10 µg protein was digested with LysC for 2 h at 37°C followed by Trypsin at room temperature overnight and protease to protein ratio of 1:100 (w/w). The digests were analyzed by nano‐liquid chromatography coupled to tandem mass spectrometry as described above loading 200 ng of protein digest. All samples were processed with MaxQuant [80] (version 2.0.1.0), with first and main peptide search tolerance set to 20, respectively 10 ppm, and MS/MS match tolerance to 40 ppm. MaxQuant's TIMS‐DDA default instrument settings were kept. Enzyme specificity was set to strict trypsin, and a maximum of three missed cleavages were allowed. Carbamidomethylation on cysteine was set as a fixed modification, methionine oxidation and protein N‐terminal acetylation as variable modifications. Protein intensities are reported as MaxQuant's Label Free Quantification (LFQ) values, as well as iTop3 [81] values (sum of the intensities of the three most intense peptides); for the latter, variance stabilization [82] was used for the peptide normalization, and missing peptide intensities, if at least two evidences exist in a group, were imputed by drawing values from a Gaussian distribution of width 0.3 centered at the sample distribution mean minus 2.8x the sample standard deviation. Imputation at protein level for both iTop3 and LFQ was performed if there were at least two measured intensities in at least one group of replicates; missing values in this case were drawn from a Gaussian distribution of width 0.3 centered at the sample distribution mean minus 2.5x the sample standard deviation. Differential expression tests were performed using empirical Bayes (moderated t‐test) implemented in the R limma package [83]. The Benjamini and Hochberg [84] method was further applied to correct for multiple testing. The criterion for statistically significant differential expression is that the maximum adjusted *p* value for large fold changes is 0.05, and that this maximum decreases asymptotically to 0 as the |Log_2_FC| of 1 is approached (with a curve parameter of one time the overall standard deviation). Proteins consistently significantly differentially expressed through 20 protein imputation cycles were subsequently flagged.

### 5.21 Proteomics Analysis

Proteins derived from iMNs with |Log_2_FC| of ± 1.5 were considered up or downregulated in comparison to their control. R studio custom routine was used for further analysis and comparison between groups and to generate heatmaps of protein expression. To generate heatmaps proteomics data were analyzed in R using the *gplots* package. The dataset was imported from a CSV file, and numeric columns corresponding to protein abundance values and |Log_2_FC| changes were extracted to construct a data matrix. Hierarchical clustering of rows was performed using Euclidean distance and complete linkage to assess similarity in protein expression patterns. Heatmaps were generated with the *heatmap.2* function, applying a diverging color scale ranging from blue (negative fold change) to white (no change) to red (positive fold change). To generate Volcano plots proteomics data were analyzed and visualized in R using the *ggplot2* and *ggrepel* packages. The dataset, containing protein identifiers, |Log_2_FC| values, and corresponding *p* values, was imported from a CSV file. Column names were standardized, and both |Log_2_FC| and *p* values were converted to numeric format. Volcano plots were generated by plotting |Log_2_FC| on the x‐axis and –Log_10_(p) on the y‐axis. Data points were color‐coded according to significance (red = significant, black = not significant) and scaled by |Log_2_FC| magnitude. A dashed horizontal line was added at –Log_10_(0.05) to indicate the significance threshold.

For Gene ontology analysis of iMNs and *C9‐500* animals’ proteome, ShyniGO 0.82 was used (https://bioinformatics.sdstate.edu/go/). The FDR cutoff was set to 0.01 and the minimum pathway size was set to 2. Enriched pathways were further plotted using GraphPad Prism version 9. The human protein atlas (https://www.proteinatlas.org/) was used to manually annotate proteins in Figure 3 and Figure S4.

### 5.22 Enzyme‐Linked Immunosorbent Assays (ELISA)

ELISA kits from Abcam were used to detect the expression levels of inflammatory cytokines IL‐1β (Abcam, ab197742), IL‐6 (Abcam, ab222503), and TGF‐β1 (Abcam, ab277719), liver function Aspartate Aminotransferase ((AST), Abcam, ab263882) Alanine aminotransferase ((ALT), Abcam, ab282882) in the serum of *WT* and *C9‐500* pre and post TLN treatment. The experimental procedures were followed according to the kit instructions, including the generation of standard curves. Based on the generated standard curves, the concentrations of various inflammatory cytokines in the samples were calculated. The Urea Nitrogen (BUN) colorimetric detection kit was from ThermoFischer Scientific (EIABUN) and the procedure was followed according to the kit instructions, based on the standard curves, the concentration of serum urea was calculated.

### 5.23 Muscle Histochemistry

Animals were deeply anesthetized with isoflurane and decapitated before the gastrocnemius muscles were dissected. Muscles were embedded in OCT compound (Fisher Scientific) and placed on dry ice. Sections were cut at 14 µm on a cryostat and placed directly onto coverslips. For H&E staining, muscle sections were stained with hematoxylin for 5 min, followed by three 10‐min washes in deionized H_2_O. Muscles were then placed in eosin G for 1 min, then rinsed in 70% ethanol. The tissue was then dehydrated in a series of ethanol washes (70%, 95%, and 100%), rinsed in xylene, then mounted on a glass slide. NADH staining was performed by incubating muscle sections in 0.2 M Tris buffer containing Nitrotetrazolium Blue (Sigma) and β‐nicotinamide adenine dinucleotide (NADH, Sigma) for 30 min at 37°C. Following three washes in deionized H_2_O, coverslips were mounted onto a glass slide and imaged using an Olympus microscope (BX‐51).

### 5.24 Measurement of Muscle Coordination and Survival

Rotarod apparatus (Ugo Basile, Comerio, Italy) was used to assess general motor performance during the light phase of the 12 h light/12 h dark cycle. For the inverse rotarod, the rod accelerated from 15 to 33 rpm in 10 s, 33 rpm to 15 rpm in 10 s, followed by inversing the direction of the rod and repeating the same procedure. One trial lasted a maximum of 110 s, with 3 min rest in between trials. Three trials every 10 days were performed *for C9‐500* animals and respective controls. Mice were habituated on the rotating rod at fixed speed for 3 days prior to the baseline recording. All measurements were performed blinded to the genotype of the mice. The hanging wire test was done by placing the mice on top of a cage cover and once the animal was stable, the cage cover was gently inverted, and the latency of the first fall was recorded. The average number of falls was assessed within 120 s. To measure longevity, the mice were followed until they became immobile and required food and water inside the cage.

### 5.25 Statistics

None of the animals were excluded for statistical analysis. For mass spectrometry and proteomic analysis of iMNs 3 *C9ORF72* patients’ lines two healthy individuals and one Isogenic Control were used, and for the *C9‐500* mice 3‐6 biological replicates were taken into consideration, including 3 *WT* saline and TLN‐treated as well as 3 *C9‐500* animal saline and TLN‐treated. Analyses were done using GraphPad Prism 9.0. Statistical significances were evaluated by two‐tailed, unpaired Student's t test and one‐ or two‐way ANOVA followed by post hoc correction for multiple comparisons such as Sidak, Bonferroni or Tukey test were used to evaluate statistical significance as indicated in the respective figure legend. Values are expressed as mean ± standard error of the mean (SEM). ^*^ *p* < 0.05, ^**^ *p* < 0.01, ^***^ *p* < 0.001, ^****^ *p*<0.0001 throughout the manuscript.

## Author Contributions

F.P., C.P., and S.S., conceived the study and wrote the manuscript. F.P. performed experiments and data analyses with contributions from T.D.T., S.B., A.S., R.D. Muscle histology and analyses were performed by M.S. and O.J. S,Y.N.T. performed rodent colony maintenance, serum collection and behavioral phenotyping. B.M. performed LC‐MS analyses for GM1 levels. C.P., generated and supervised liposomal formulation and S.S. supervised the overall project. C.P., S.S. and S.E. provided reagents, experimental inputs and data discussion.

## Funding

The study was funded by the start‐up InnoMedica Schweiz AG, to perform initial TLN studies and further experiments were supported by European Research Council (ERC) under the European Union's Horizon 2020 research and innovation program (grant agreement #725825), Swiss National Science Foundation (project grant #179436), Swiss Foundation for Research on Muscle Diseases, and E‐rare grant (CALSER) and the Spinal Cord Injuries/Disease Research Program (SCIDRP), University of Missouri, Missouri, USA, to S.S.

## Ethics Statement

The study was approved by the Animal Commission of Canton of Bern, Switzerland, license number BE‐35/17, BE‐82/18, and ACUC #53562. Cells were anonymized and provided under an MTA with the respective consortia.

## Conflicts of Interest

The authors declare no conflict of interest.

## Supporting information




**Supporting File 1**: advs73639‐sup‐0001‐SuppMat.docx.

**Supporting File 2**: advs73639‐sup‐0002‐Data.zip.

## Data Availability

The data that support the findings of this study are available from the corresponding author upon reasonable request.
