# Supplementary material for: Engineered GM1 Intersects Between Mitochondrial and Synaptic Pathways to Ameliorate ALS Pathology
Source: Adv Sci (Weinh). 2026 Jan 5;13(20):e14128. doi: 10.1002/advs.202514128 (PMC13067844; doi:10.1002/advs.202514128)
Supplement: Supplementary file 1 — Supporting File 1: advs73639‐sup‐0001‐SuppMat.docx. [file ADVS-13-e14128-s002.docx]

((Supporting Information can be included here using this template))

Supporting Information

Engineered GM1 Intersects Between Mitochondrial and Synaptic Pathways to Ameliorate ALS pathology

*Federica Pilotto^3,7^, Tristan Dellazizzo Toth^1,2^, Silvano Bond^1,2^, Alexander Schmitz^7^, Rim Diab^7^, Sara Y. Ngo Tenlep^1,2^, Brian Mooney^4,5^, Silvia Erni^6^, Martina Schobesberger^7^, Olivier Scheidegger^7^, Camille Peitsch^6^, Smita Saxena^1,2,7,*^*

^1^ Department of Physical Medicine & Rehabilitation, University of Missouri, Columbia, MO, USA

^2^ NextGen Precision Health, University of Missouri, Columbia, MO, USA

^3^ Institut Neuromyogène, Pathophysiology and genetics of the neuron and muscle, Inserm U1315, CNRS, Université Claude Bernard Lyon I, UMR 5261, Lyon, France.

^4^Division of Biochemistry, University of Missouri, Columbia, MO, USA

^5^Charles W Gehrke Proteomics Center, University of Missouri, Columbia, MO, USA

^6^ InnoMedica Schweiz AG, Bern, Switzerland

^7^ Department of Neurology, Inselspital University Hospital, Bern, Switzerland.

*Corresponding Author:

Smita Saxena,

Email: smitasaxena@health.missouri.edu

Email


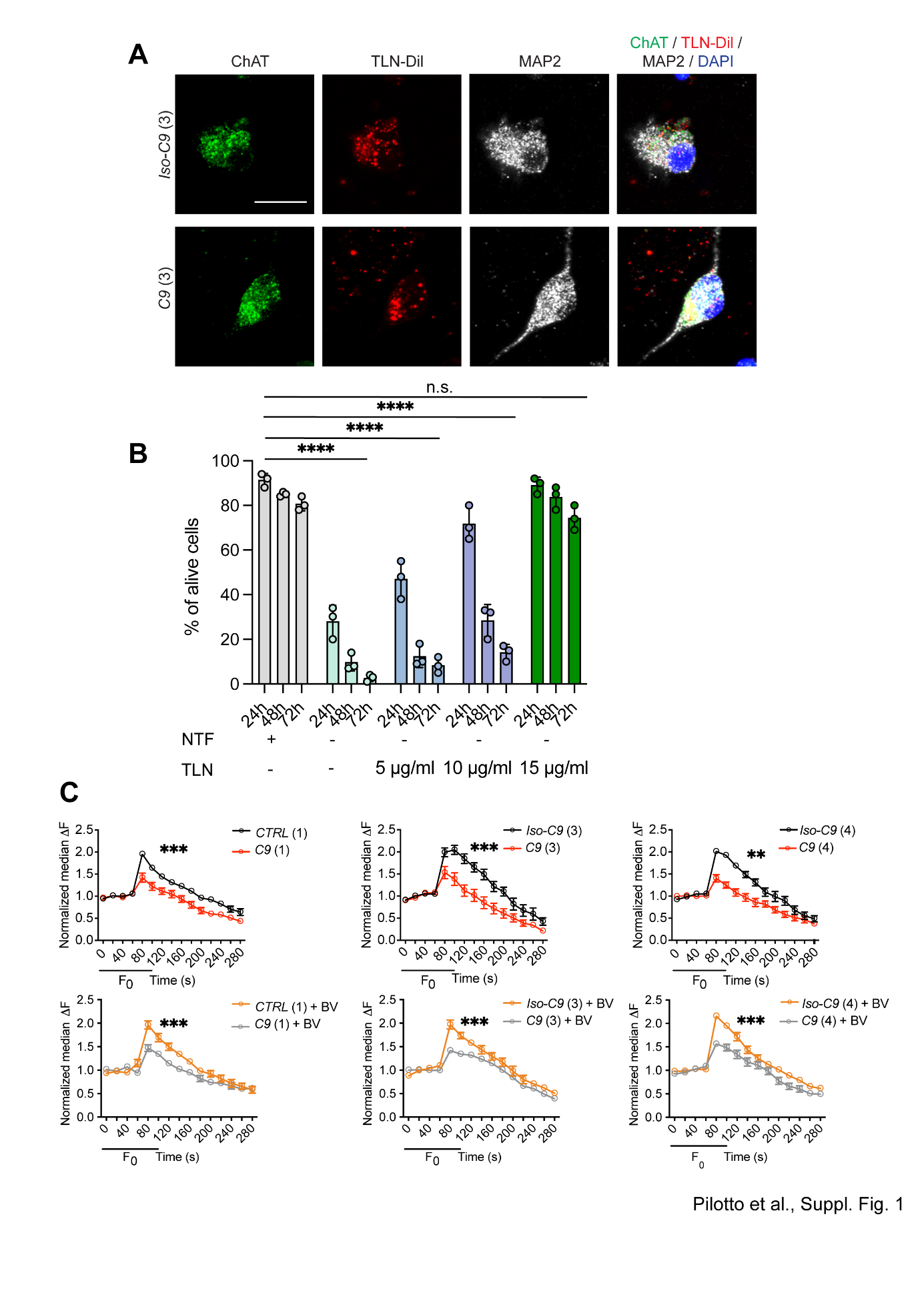


**Supplementary Figure 1.**

(**A**) Representative confocal images of 1 week old iMNs from *iso-C9* (3) and *C9* (3) patient lines illustrating the uptake of TLN conjugated with Dil. Scale bar: 20μm. Note the vesicular pattern of internalized TLN after 24 h of TLN administration.

(**B**) Quantification of the percentage of viable cells at 24 h, 48 h, and 72 h under different treatment conditions. 10 days in vitro (DIV) iMN cultures maintained with neurotrophic factors (NTF+) show high survival across all time points. Removal of NTF (NTF-) results in rapid and progressive loss of cell viability. Treatment with TLN at 5 µg/ml, 10 µg/ml, or 15 µg/ml partially or fully rescues cell survival in a dose-dependent manner, with 15 µg/ml restoring viability to levels comparable to NTF-treated controls. Two-way ANOVA: Time points(F2,30)=133.4****, Treatment(F4,30)=390.8****, interaction(F8,30)=12.88****; Šidák’s post hoc statistical significance reported in the figure: **** *p* < 0.0001; n.s., not significant.

(**C**) Calcium imaging traces of individual Control (*CTRL*), and Isogenic control lines with corresponding *C9* patients’ iMNs lines. Note how TLN is able to restore mitochondrial calcium transients compared to GM1 alone. Multiple t test at 100s: *CTRL* (1) mean: 1.639; *C9* (1) mean: 1.224, *p* value: 0.004; *C9* (1) mean: 1.224; *C9* (1) + BV mean: 1.340, *p* value: 0.30; *Iso-C9* (3) mean: 1.923; *C9* (3) mean: 1.250, *p* value <0.0001; *C9* (3) mean: 1.250; *C9* (3) + BV mean: 1.339, *p* value: 0.27; *Iso-C9* (4) mean: 2.046; *C9* (4) mean: 1.384, *p* value: 0.001; *C9* (4) mean: 1.384; *C9* (4) + BV mean: 1.484, *p* value:0.56.

**
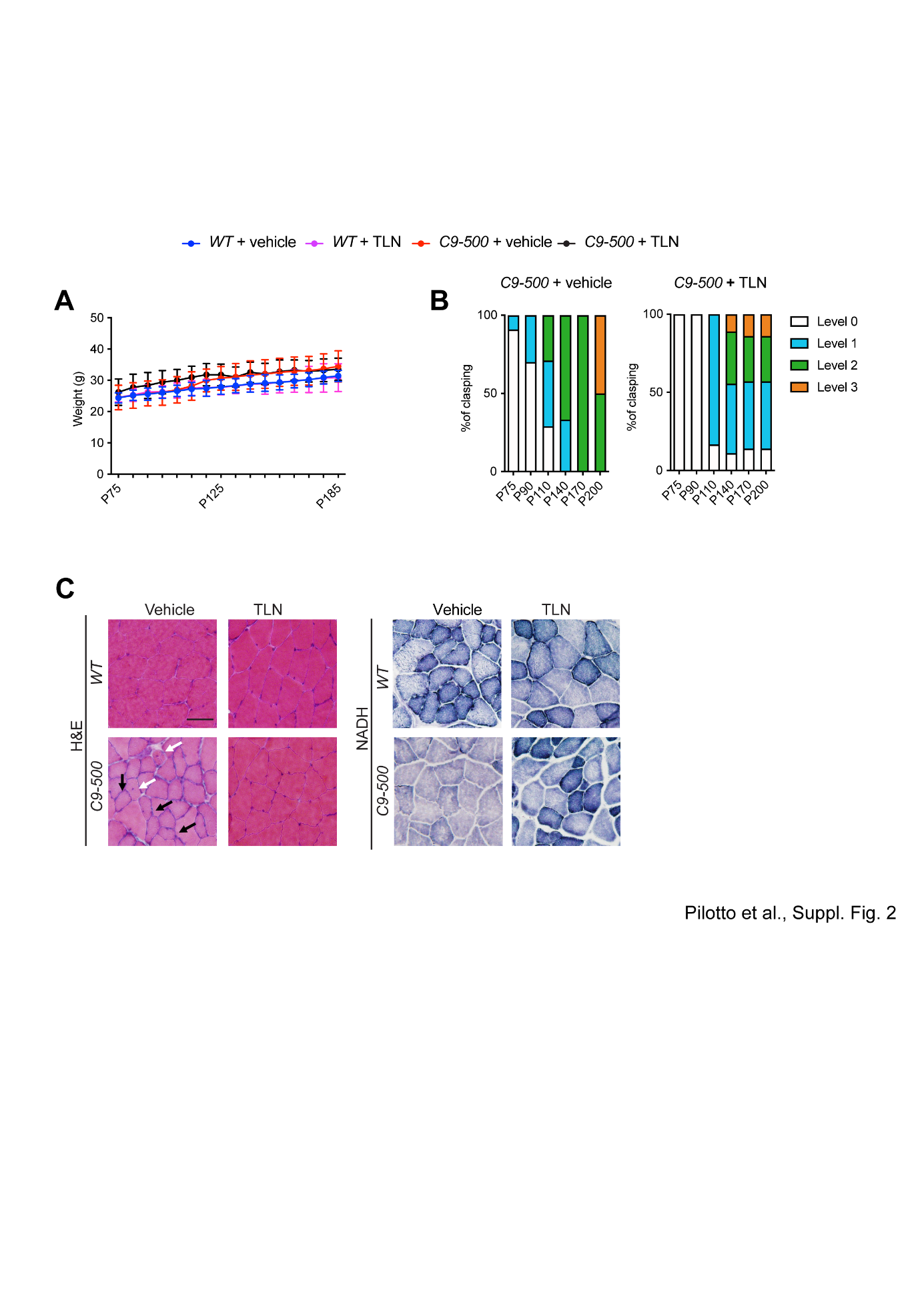
Supplementary Figure 2.**

(**A**) TLN administered in drinking water at 7.5mg kg does not affect the weight of the *WT* and *C9-500* animals.

(**B**) TLN ameliorates the clasping phenotype observed in *C9-500* animals.

(**C**) H&E staining revealed angulate fibers and the presence of central nuclei in *C9-500* + vehicle treated animals, which are not observed in *C9-500* animals that received TLN. NADH staining revealed a checkerboard pattern in *WT* muscle that is completely lost in *C9-500* animals treated with vehicle whereas *C9-500* mice treated with TLN display a conserved checkerboard pattern. Scale bar: 100μm.


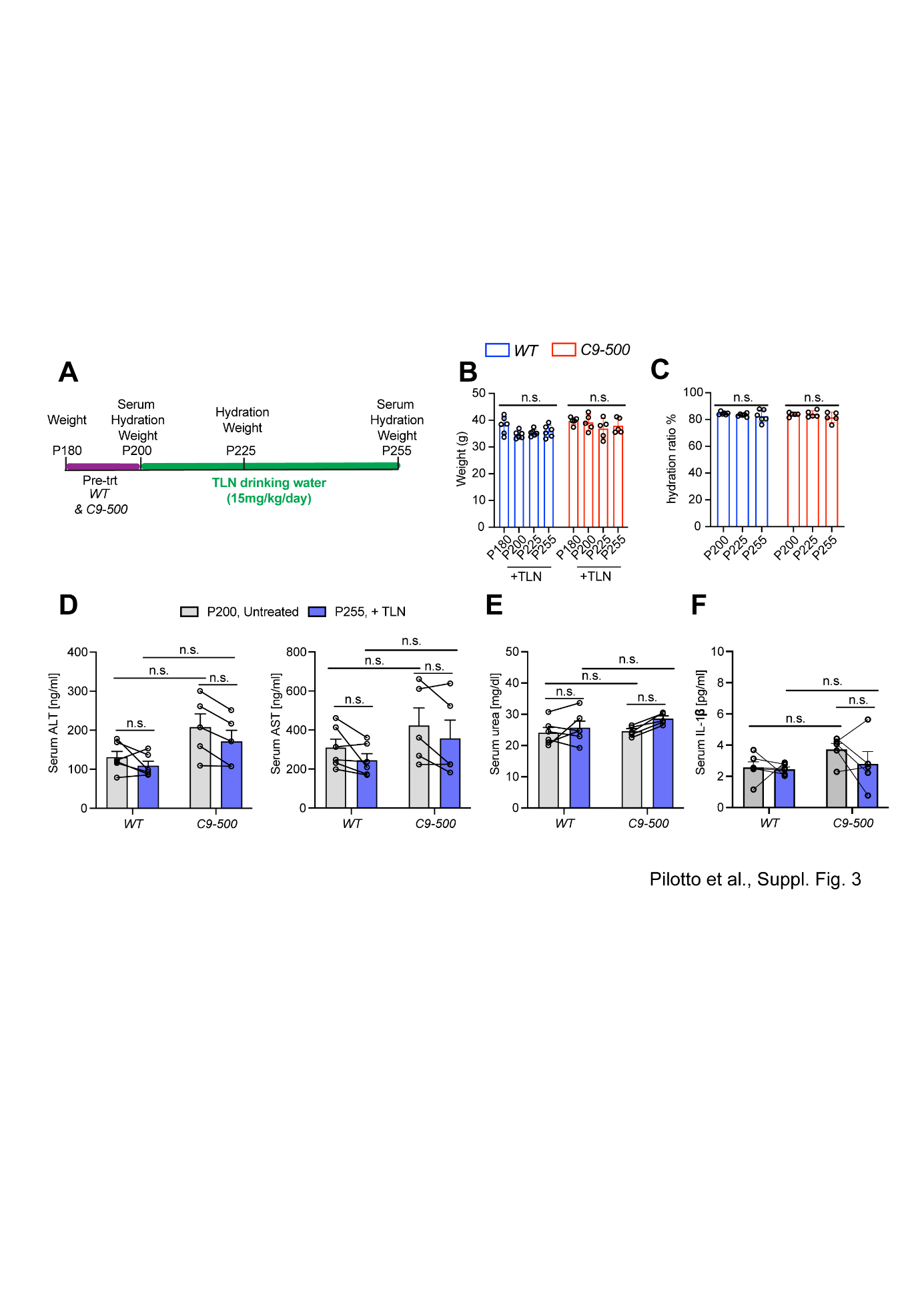


**Supplementary Figure 3.**

(**A**) Schematic showing the experimental design. Late-symptomatic *C9-500* mice were treated with TLN; 15 mg/kg/day. Serum was longitudinally collected at P200 (pre-treatment) and at P255 (post-treatment) from the same mice.

(**B**) Body weight measured pre- and post-TLN treatment in *WT* and *C9-500* mice. TLN administration did not significantly affect body weight at any time point.

(**C**) Hydration ratio was measured via EchoMRI pre-treatment and during TLN treatment. No differences were observed between groups or across treatment time-points, indicating preserved hydration status during TLN treatment via drinking water.

(**D**) Serum markers of liver integrity at P200 (untreated, grey) and at P255 after TLN treatment (blue). Serum ALT levels (left) and AST levels (right) were modestly elevated in untreated *C9-500* mice compared to *WT* (Two-way ANOVA: treatment n.s.; genotype * *p*=0.007, interaction n.s.; Šidák’s multiple comparison test n.s. reported in the figure), but TLN treatment did not exacerbate ALT or AST levels in either genotype.

(**E**) Serum urea levels at P200 (pre-treatment) and P255 (+TLN). No significant changes were detected between *WT* and *C9-500* mice or following TLN treatment, indicating absence of kidney toxicity. Data are shown as mean ± SEM; individual mice are plotted.

(**F**) Serum probed for pro-inflammatory cytokine IL-1 β at P200 (pre-treatment) and P255 (+ TLN) revealed no further increase in inflammatory cytokines. Two-way ANOVA: treatment n.s.; genotype n.s., interaction n.s.; Šidák’s multiple comparison test n.s. reported in the figure.

**
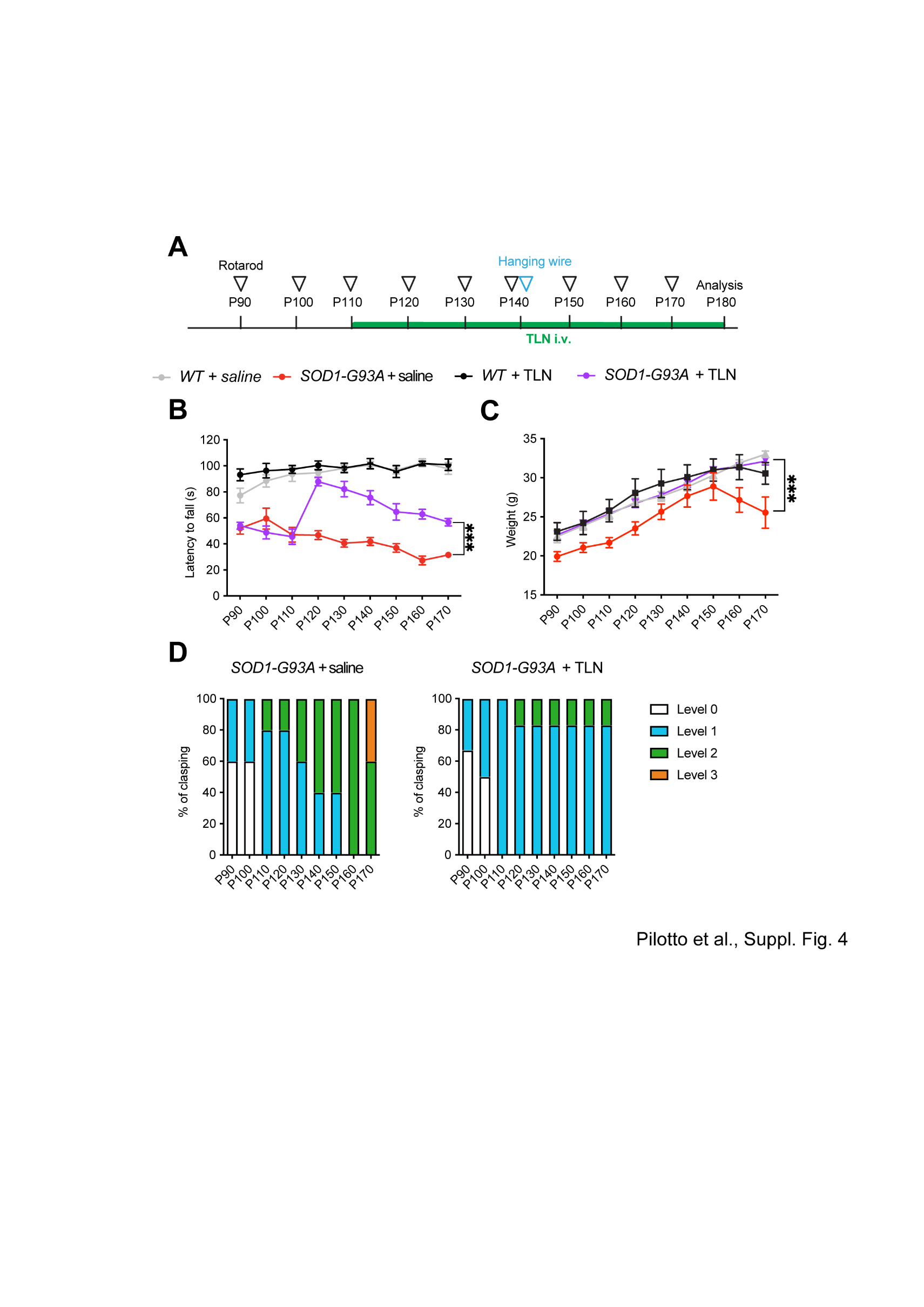
**

**Supplementary Figure 4.**

(**A**) Schematic timeline of i.v. TLN administration in *SOD1-G93A* animals. TLN was administered at a dosage of 15 mg/kg every alternate day.

(**B**) Inverse rotarod presented as latency to fall. TLN-treated *SOD1-G93A* animals show significant improvement in motor performance compared to saline controls. Two-way ANOVA interaction F(24,160)=5.733, *p* value <0.0001, time F(5.6,112.5)=4.880, *p* value =0.0003, genotype F(3,20)=247.2, *p* value <0.0001. Tukey’s multiple comparisons: P110 *SOD1-G93A* + saline vs *SOD1-G93A* + TLN n.s.; P120 *SOD1-G93A* + saline vs *SOD1-G93A* + TLN ***; P130-P140 *SOD1-G93A* + saline vs *SOD1-G93A* + TLN **; P150 *SOD1-G93A* + saline vs *SOD1-G93A* + TLN *; P160-P170 *SOD1-G93A* + saline vs *SOD1-G93A* + TLN ***. n= 6 *WT* + saline, 6 *SOD1-G93A +* saline, 6 *WT* + TLN, 6 *SOD1-G93A* + TLN.

(**C**) *SOD1-G93A* + saline animals show a significant weight loss from P150, on the contrary *SOD1-G93A* animals treated with TLN do not show a similar decline in weight. ***. n= 6 *WT* saline, 6 *SOD1-G93A* saline, 6 *WT* + TLN, 6 *SOD1-G93A* + TLN.

(**D**) *SOD1-G93A* animals treated with TLN significantly ameliorate their clasping phenotype compared to saline treated controls. ***. n= 6 *WT* + saline, 6 *SOD1-G93A* + saline, 6 *WT* + TLN, 6 *SOD1-G93A* + TLN.

**
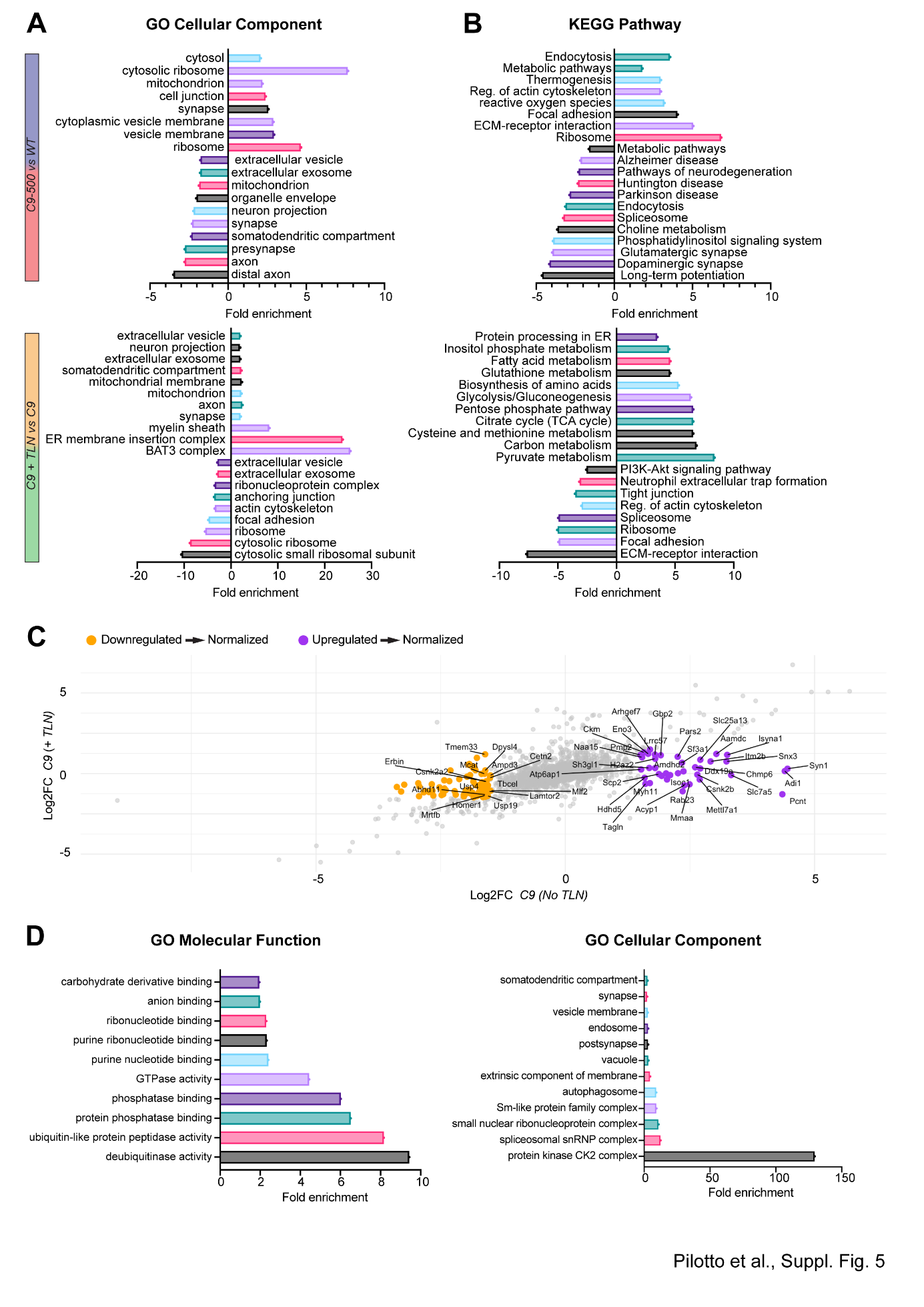
**

**Supplementary Figure 5.**

**(A-B)** Bar plot illustrating gene ontology (GO) cellular component and KEGG pathway analysis of protein up and downregulated in *C9-500* animals vs *WT* and after TLN administration.

(**C**) Dot plot highlighting proteins that were up (violet dots) or downregulated (orange dots) in *C9-500* animals at baseline that returned to normal levels after TLN treatment considering a |Log₂FC| of ± 1.5.

(**D**) GO analysis of molecular function and cellular components of differentially expressed proteins (DEPs) that become comparable to WT expression levels after TLN treatment (**C**), highlighting normalization of proteins involved in autophagy and synaptic metabolism.

**
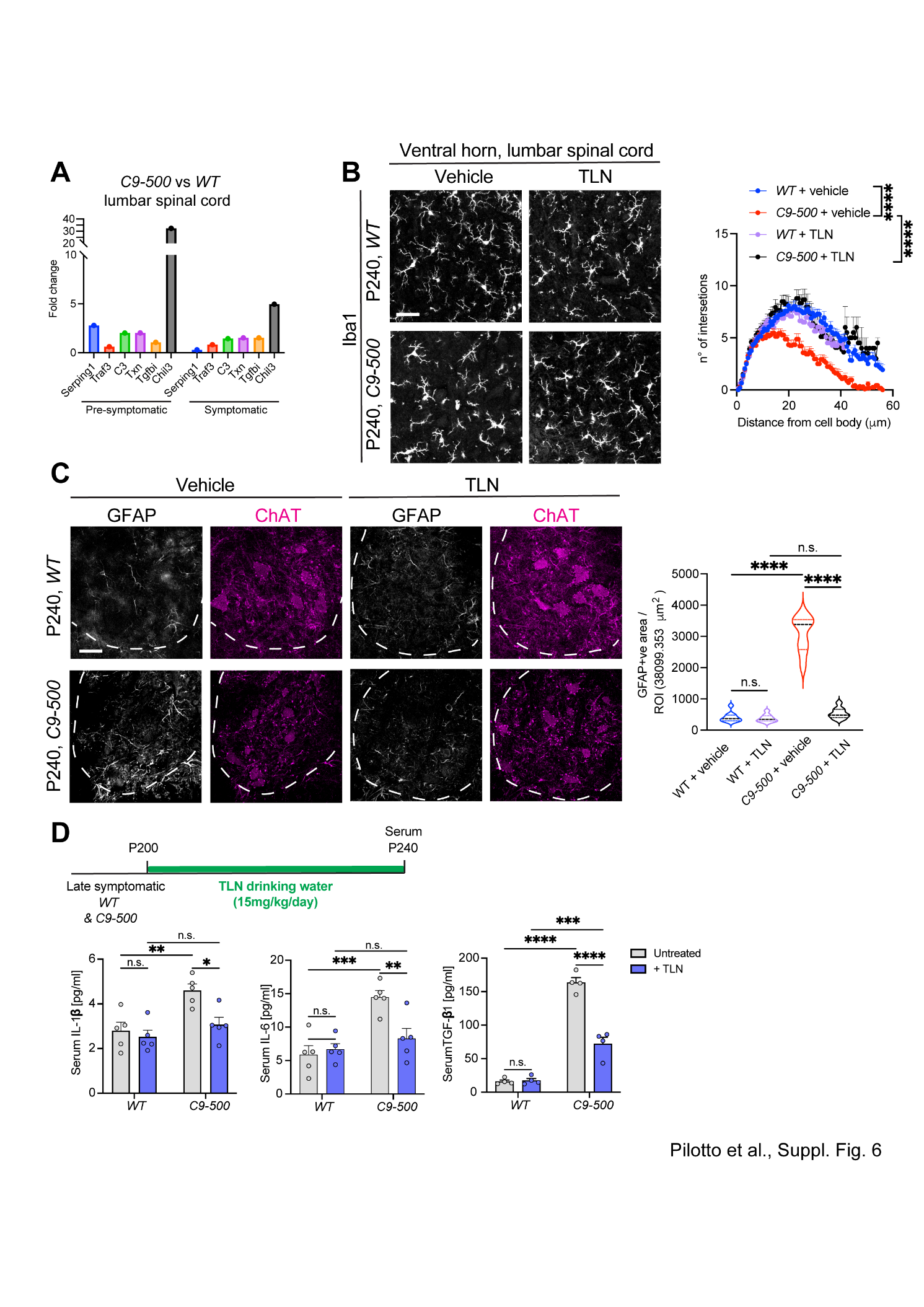
Supplementary Figure 6.**

**(A)** Heat maps illustrate fold change of differentially expressed proteins involved in inflammatory pathways in the *C9-500* spinal cord. Proteomic analyses were performed on *C9-500* animals at P110 (pre-symptomatic stage) and P180 (symptomatic stage).

**(B)** Representative images of Iba1⁺ microglia in the ventral horn from *WT* and *C9-500* mice treated with vehicle or TLN. Sholl analysis revealed reduced microglial ramification in *C9-500* saline mice, which was restored by TLN treatment in drinking water. Right: quantification of the number of intersections as a function of distance from the soma. One-way ANOVA F=42, *p*<0.0001, Šidák’s multiple comparison test: *WT* + vehicle vs *WT* + TLN n.s.; *WT* + vehicle vs *C9-500* + vehicle ****; *C9-500* + vehicle vs *C9-500* + TLN ****.

**(C)** Representative images of GFAP/ChAT co-staining in *WT* and *C9-500* mice under vehicle or TLN treatment. Right: quantification of GFAP⁺ occupied area within a defined ROI (38,099.353 μm²) in the ventral horn. TLN significantly reduced GFAP⁺ area in *C9-500* mice. One way ANOVA F=160, *p*<0.0001, Šidák’s multiple comparison test: *WT* + vehicle vs *WT* + TLN n.s.; *WT* + vehicle vs *C9-500* + vehicle ****; *C9-500* + vehicle vs *C9-500 +* TLN ****.

Scale bars (**A & B**): 50 μm. Data are shown as mean ± SEM.

**(D)** *C9-500* and *WT* mice were treated with 15 mg/kg of TLN in drinking water from P200 to P240. Serum was collected at P240 and probed for IL-1 β, IL-6, and TGF-β1. Age matched untreated *C9-500* animals present elevated levels of IL-1β, IL-6 and TGF-β1 which are significantly reduced after TLN treatment, whereas TLN does not affect *WT* animal’s circulating cytokines. IL-1β: one-way ANOVA F=8.271, *p*=0.0015, Šidák’s multiple comparison test *WT* vs *WT* + TLN n.s., *C9-500* vs *C9-500* + TLN *, *WT* vs *C9-500* untreated **, *WT* + TLN vs *C9-500* + TLN n.s.; IL-6: one-way ANOVA F=10.73, *p*=0.0004, Šidák’s multiple comparison test *WT* vs *WT* + TLN n.s., *C9-500* vs *C9-500* + TLN **, *WT* vs *C9-500* ***, *WT* + TLN vs *C9-500* + TLN n.s.. TGF-β1: one-way ANOVA F=114.1, *p*<0.0001, Šidák’s multiple comparison test *WT* vs *WT* + TLN n.s., *C9-500* vs *C9-500* + TLN ****, *WT* vs *C9-500* ****, *WT* + TLN vs *C9-500* + TLN ***. N=3 animals/genotype/treatment for immunofluorescence and 5 animals/genotype/treatment for ELISA assay.
